# Retrieve-Then-Verify for Evaluating Evidence Support and Hallucination in Large Language Model–Generated Medical Information: Empirical Study

**DOI:** 10.2196/93761

**Published:** 2026-09-16

**Authors:** Zhaohui Liang, Cynthia Sheffield, Gisela Butera, Sameer Antani

**Affiliations:** 1 Division of Intramural Research National Library of Medicine National Institutes of Health Bethesda, MD United States; 2 NIH Library National Institutes of Health Bethesda, MD United States

**Keywords:** AI, large language models, digital health, medical informatics, evidence-based medicine, information quality, information retrieval, hallucinations, systematic review, risk of bias

## Abstract

**Background:**

Despite high reported accuracy on clinical and evidence appraisal tasks, AI-generated medical information may lack explicit support from source documents. This creates challenges for digital health practitioners regarding transparency, auditability, and trust when AI systems are used for evidence synthesis, guideline development, and clinical knowledge management. Large language models (LLMs) can generate fluent and seemingly correct outputs, but existing evaluations often rely on agreement with human judgments and do not directly assess whether AI-generated content is grounded in underlying evidence.

**Objective:**

This study measures evidence support and hallucination in AI-generated medical information by assessing the extent to which LLM-generated risk-of-bias assessments are supported by source clinical trial reports.

**Methods:**

We evaluated 3 LLMs (GPT-5, OpenAI o3-mini, and GPT-3.5) on risk-of-bias (RoB 2) assessment using all 97 randomized controlled trials for which the source Cochrane systematic review provided complete human RoB 2 annotations and full-text reports were accessible, constituting the complete reference set. No train-validation split was applied; all 97 studies were used for evaluation. Model outputs were constrained to structured RoB 2 signaling questions and domain-level judgments. For each generated claim, relevant text passages were retrieved from trial reports using the Okapi BM25 (Best Matching 25) algorithm. A verification step assigned evidence verdicts (supported, contradicted, not found, or out of scope) with verbatim quotations. We quantified evidence support rates and conservative and strict hallucination rates. Task performance was evaluated using exact and binary accuracy, sensitivity, specificity, F1-score, Youden J, and agreement with human reviewers using Cohen κ and Fleiss κ.

**Results:**

Binary accuracy of AI-generated risk-of-bias judgments was high across domains (90%-98%), whereas exact accuracy was substantially lower (42%-71%), reflecting frequent disagreements in severity classification despite correct directional classification. GPT-5 achieved the strongest overall performance, including perfect binary accuracy for overall risk-of-bias conclusions and the highest agreement with human reviewers (quadratic κ up to 0.81). However, evidence support rates across models ranged from only 60% to 65%, with conservative hallucination rates of 34%-37%. GPT-5 showed the highest mean evidence support (64.3%) and the lowest strict hallucination rate (35.7%). Mean top-1 BM25 retrieval scores were similar across models (approximately 30-31), suggesting that differences in hallucination were not primarily attributable to differences in retrieval strength.

**Conclusions:**

AI-generated medical information can achieve high decision-level accuracy while still lacking documentary support in a substantial proportion of outputs. Measuring evidence support and hallucination reveals important limitations that are not captured by agreement metrics alone. Retrieval-based evidence verification provides a reproducible and transparent approach for evaluating the reliability of AI-generated medical information, with direct relevance to digital health practice, evidence-based medicine, and medical informatics.

## Introduction

Clinical decision support systems, evidence synthesis platforms, and clinical knowledge management tools increasingly incorporate AI-generated medical information to assist clinicians, researchers, and health system stakeholders. In many digital health applications, large language models (LLMs) are used to summarize clinical evidence, draft guideline text, support systematic reviews, or provide contextual explanations at the point of care. These systems promise efficiency and scalability, but they also introduce new challenges related to transparency, auditability, and trust when AI-generated outputs are used to inform clinical and policy decisions.

LLMs have demonstrated strong performance across a wide range of biomedical knowledge and reasoning tasks, accelerating their adoption in health care workflows [1-6]. However, as AI-generated medical information moves from experimental settings into routine digital health infrastructures, ensuring that AI-generated content is reliable and explicitly grounded in the source evidence has become a central concern for clinicians, informaticians, and digital health application developers. In clinical and evidence-based contexts, plausible but unsupported statements may influence decision-making, undermine user trust, and pose risks to patient safety.

A growing body of literature indicates that LLMs can produce hallucinations, defined here as generated outputs that are not supported by source materials, raising concerns regarding information quality and safety in health care applications [7-12]. These risks have been documented across multiple settings relevant to digital health practice, including fabricated or inaccurate references in systematic reviews [7,9], unsupported statements in clinical text summarization [8], and acceptance or elaboration of fabricated clinical details embedded in prompts during clinical decision support [11]. Conceptual and sociotechnical analyses further suggest that naive reliance on AI-generated medical information may amplify automation bias, erode professional expertise, and reinforce self-referential learning loops in digital health systems [10]. Collectively, these studies establish hallucination as a pervasive information quality risk while also highlighting substantial heterogeneity in how it is defined, measured, and evaluated.

Despite growing recognition of these risks, most evaluations of hallucination in AI-generated health information rely on indirect measures such as agreement with human judgments, task-level accuracy, or expert preference ratings. Although useful, these metrics provide limited insight into whether the generated content is supported by the underlying evidence. Semantically correct outputs may be penalized because of wording differences, whereas fluent but unsupported statements may be judged acceptable. For digital health systems deployed in clinical and evidence-based settings, this represents a critical blind spot: agreement with human labels does not guarantee that AI-generated medical information can be transparently traced back to authoritative source documents.

This limitation is particularly consequential in evidence-based medicine, where clinical decisions depend on explicit and verifiable links between conclusions and underlying evidence. Evidence synthesis requires not only summarization but also careful appraisal of study design, conduct, and reporting. Within systematic reviews, the Cochrane risk-of-bias (RoB) assessment tool formalized a structured, domain-based approach to judgments anchored in trial methodology rather than undifferentiated quality scores [13]. However, RoB assessment has long been recognized as labor intensive, variably reproducible, and prone to misapplication in specific domains such as selective reporting [14-16]. Although the revised RoB 2 tool introduced outcome-specific domains, signaling questions, and explicit decision rules to improve consistency [17], the process continues to require detailed evidence collection and justification, limiting scalability in digital evidence synthesis workflows.

Recent studies have explored the use of LLMs to assist with evidence appraisal tasks, including RoB assessment, and have reported promising agreement with human judgments [18]. However, these applications also illustrate a broader challenge for AI-generated medical information: outputs that appear correct may still lack support from source documents, particularly when models infer intent, assumptions, or methodological details that are not explicitly reported. In this sense, hallucination reflects a general and high-stakes failure mode of AI-generated medical information in digital health systems, where plausible statements lack documentary support but may nonetheless influence downstream decisions.

To address this gap, we focus on measuring evidence support and hallucination at the claim level by explicitly linking AI-generated statements to their source documents. Rather than relying solely on agreement with human judgments, we operationalize hallucination as the absence of documentary support in the underlying source material. Using structured retrieval to identify relevant passages and a verification step to determine whether each generated claim is supported, contradicted, or not found in the source material, we quantify evidence support and unsupported statements across models, domains, and studies. While RoB assessment provides a concrete and well-defined test case, the proposed evaluation approach is model-agnostic and task-general. It can be applied broadly to AI-generated medical information in clinical decision support, evidence synthesis, guideline development, and other digital health applications that require transparent evidence grounding.

By reframing hallucination as a problem of information grounding rather than disagreement with human judgments, this study addresses a central challenge in the evaluation of AI-generated medical information. The proposed approach provides reproducible, source-linked quality metrics that are directly relevant to digital health applications. As LLMs are increasingly integrated into clinical, research, and policy workflows, evaluation methods that make evidentiary support explicit will be essential for promoting transparency, accountability, and the safe integration of AI into evidence-based medicine and medical informatics.

The objective of this study was to develop and evaluate a retrieve-then-verify (RtV) framework for measuring evidence support and hallucination in LLM-generated medical information at the claim level. Specifically, we applied this method to RoB assessments generated by 3 LLMs across 97 randomized controlled trials from a Cochrane systematic review. The study aimed to quantify the proportion of model outputs that agreed with human judgments yet lacked explicit evidence support in the underlying trial literature.

## Methods

### Study Design and Data Sources

We conducted a retrospective evaluation to assess evidence support and hallucination in AI-generated medical information, using RoB assessment as a representative evidence appraisal task. The study design evaluated whether LLMs can generate judgments that are both accurate and explicitly grounded in source documents.

The evaluation corpus was derived from a 2023 Cochrane systematic review on primary-level and community health worker interventions for the prevention of mental disorders and the promotion of well-being [19]. Human RoB assessments from the review served as the reference standard for task performance and agreement analyses. In accordance with standard Cochrane methodology, RoB assessments in the source review were performed independently by 2 reviewers using the RoB 2 tool, with discrepancies resolved through consensus discussion; full reviewer details are reported in the source review [19]. Reviewers were systematic review specialists with expertise in global mental health interventions; their institutional affiliations and authorship details are listed in the source review [19]. Of the 113 randomized trials included in the review, analyses were restricted to 97 (85.8%) studies with complete RoB data and accessible full-text reports in PDF. The included trials were published between 1996 and 2022. Assessments covered 7 outcome categories: diagnosis of mental disorders, quality of life, adverse events, psychological functioning and impairment, depressive symptoms, anxiety symptoms, and distress or posttraumatic stress disorder symptoms.

### LLMs and RoB Assessment Workflow

To generate AI-generated RoB assessments, we implemented the Cochrane RoB 2 instrument as a structured, schema-constrained Python (Python Foundation) prompt [20]. Text and tables extracted from each trial report were provided as model input. We evaluated 3 LLMs representing different model families and deployment profiles: GPT-5 (August 2025) [21], OpenAI o3-mini (January 2025) [22], and GPT-3.5 Turbo (January 2024) [23].

All models were accessed via the OpenAI API. For GPT-3.5 Turbo, default decoding parameters were used (temperature=1 and max tokens=4096). GPT-5 and OpenAI o3-mini are reasoning models for which the temperature parameter is not applicable; the maximum number of completion tokens was not explicitly set, defaulting to each model’s maximum output capacity, and reasoning effort was set to the default level of medium. Prompts and postprocessing constrained model outputs to predefined response options for RoB 2 signaling questions and domain-level judgments. Outputs were returned in structured JSON format, enabling automated validation, reproducible analysis, and downstream evaluation. This constrained design minimized variability associated with free-text generation and ensured consistency across models and studies. The overall workflow is illustrated in Figure 1.

**Figure 1:**
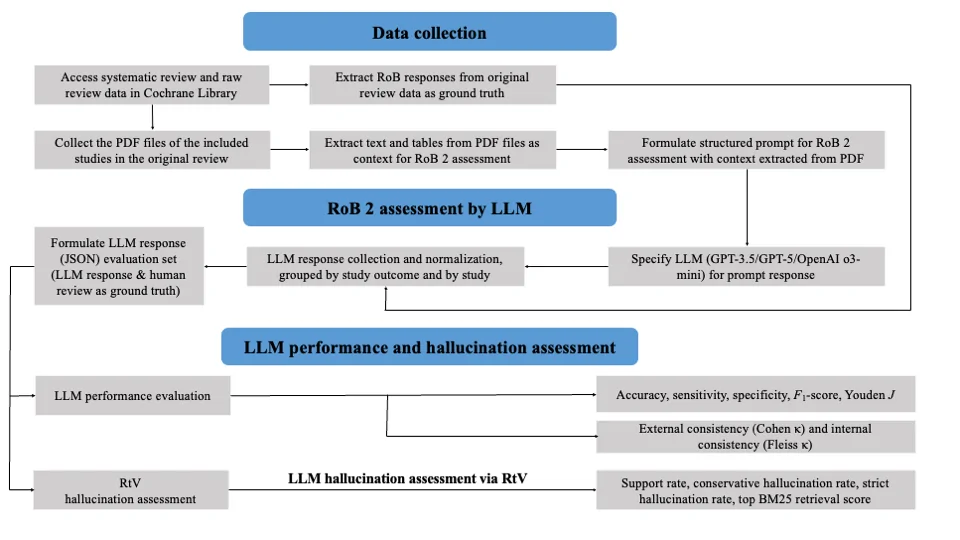
Flowchart of AI-generated medical information assessment. LLM: large language model; RoB: risk of bias; RtV: retrieve-then-verify.

### Prompt Engineering and Output Standardization

We adopted a structured prompting strategy to standardize RoB 2 assessments and support reproducible evaluation. Prior studies have applied LLMs to RoB assessment using free-text or interactive prompting in general-purpose systems [18,24-27]. By contrast, our approach imposed strict schema constraints aligned with RoB 2 signaling questions and decision rules.

Each model output included the item identifier, item-level response, domain-level judgment, and a brief rationale consisting of a comment and explanation. This structure enabled consistent mapping to analytic variables, automated calculation of performance metrics, and traceability for adjudication. Prompt specifications included task instructions, bounded trial-report context, RoB 2 signaling questions and domain logic, instructions to base each assessment on the trial report and provide a written justification consistent with the RoB 2 decision rules, and explicit output-format requirements.

For transparency and reproducibility, the prompt engineering algorithm for structured RoB 2 responses is provided in Multimedia Appendix 1, and an example of a RoB 2 prompt and response is provided in Multimedia Appendix 2. The full prompt templates, system instructions, and JSON schemas are additionally available as literal prompt definitions in the analysis code, which may be accessed as described in the “Data Availability” statement. Prompt specifications were developed based on the structure and decision logic of the Cochrane RoB 2 instrument [20] and were not validated on a separate held-out prompt development dataset. Prompt adequacy was verified through iterative review against the instrument’s signaling questions and decision rules before full-scale application across all 97 studies.

### Task Performance and Agreement Metrics

We evaluated model outputs using complementary measures of task performance and reliability. Task performance was assessed relative to the human reference standard using exact and binary accuracy. Item-level responses were encoded on an ordinal scale (yes=5; probably yes=4; probably no=3; no=2; no information=1; and not applicable=0), and domain-level judgments were encoded as high risk=3, some concerns=2, and low risk=1.

Exact accuracy was defined as the proportion of outputs that exactly matched the human labels. Binary accuracy was computed after collapsing ordinal categories to reflect decision-level correctness. Additional performance metrics included sensitivity, specificity, *F*_1_-score, and Youden *J* statistic (sensitivity + specificity − 1).

Agreement analyses quantified reliability rather than correctness. External agreement between each LLM and human reviewers was measured using Cohen κ with linear and quadratic weights to account for the severity of ordinal disagreement. Internal agreement among LLMs was assessed using Fleiss κ. While these metrics summarize alignment with human judgments, they do not assess whether model rationales are supported by the source documents. To address this limitation, we applied an evidence-grounded, retrieval-based evaluation, which is described below.

### Retrieval-Based Evidence Support and Hallucination Assessment

For each study, full-text reports were segmented into overlapping text chunks and indexed using the Okapi BM25 (Best Matching 25) information retrieval algorithm [28]. Each chunk contained 1400 characters, with an overlap of 200 characters between consecutive chunks. The chunk size was selected to balance sufficient local context per passage with retrieval precision; the 200-character overlap helped ensure that claims spanning chunk boundaries were not missed. These parameters were applied consistently across all 97 studies and all 3 LLMs.

For each RoB 2 signaling question, a retrieval query was constructed by concatenating the question text, the model’s response, and its explanation. BM25 was used to rank document chunks and return the top-k passages (default k=5). BM25 scores were computed using the standard formulation (equation 1). As a retrieval diagnostic, we report the mean top-1 BM25 score alongside evidence support and hallucination rates.



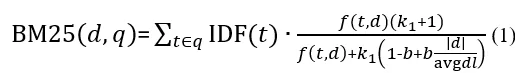



To complement lexical retrieval, we added an embedding-based semantic grounding diagnostic. For each item, the LLM explanation text was compared with each BM25-retrieved snippet using the pretrained sentence encoder sentence-transformers/all-MiniLM-L6-v2 [29]. The explanation and retrieved snippets were embedded with the same encoder, mean-pooled over token embeddings, L2-normalized, and compared using cosine similarity. This directly evaluates whether the model’s stated rationale is semantically close to the retrieved trial text, including when the supporting evidence is paraphrased rather than lexically matched.

For each item, we retained the per-snippet cosine similarities, the maximum semantic similarity score, the mean top-k semantic similarity score, and the rank of the best-matching retrieved snippet. Higher semantic similarity indicates stronger semantic grounding of the explanation in the retrieved evidence, whereas low similarity across retrieved snippets indicates weak grounding and potential hallucination risk. These embedding-based scores were used as retrieval and grounding diagnostics alongside BM25; final evidence support and hallucination labels remained based on the structured verification verdicts. The algorithm is presented in Multimedia Appendix 3. To assess whether low semantic similarity co-occurred with retrieval failure, item-level semantic similarity was additionally stratified by verification verdict and compared between supported and nonsupported items using the Mann-Whitney *U* test; the item-level association between semantic similarity and BM25 top-1 score was quantified using the Spearman rank correlation (Multimedia Appendix 4).

Verification was performed by a constrained judge LLM (GPT-5). The judge received the trial identifier, question text, model output, explanation, and retrieved passages and returned structured JSON containing 1 of 4 verdicts: supported, contradicted, not found, or out of scope. Each response also included the verdict label, a verbatim quotation drawn from the retrieved trial text that grounds the judgment, and a 1-sentence rationale, making the evidentiary basis for each item-level decision directly traceable without requiring downstream re-derivation. Free-text responses were normalized to these canonical labels.

Evidence support was defined as a supported verdict. Hallucination was operationalized as a lack of evidence support in the retrieved context. We computed 2 hallucination rates: a conservative rate counting contradicted or not found verdicts, and a strict rate additionally including out-of-scope verdicts. Metrics were summarized overall and by RoB 2 domain, and retrieval strength was examined using mean top-1 BM25 scores stratified by verdict. The RtV workflow with BM25 retrieval and LLM verdicts is provided in Multimedia Appendix 5, and an example RtV prompt and verification response is provided in Multimedia Appendix 6. An example of the full verification rationale, including BM25 retrieval scores, retrieved passages, and verdict justification for all RoB 2 signaling questions and domain-level conclusions, is provided in Multimedia Appendix 7.

### Computational Environment

All experiments were conducted using Python (version 3.10) on a Linux-based computing environment. Core libraries included numpy, pandas, scikit-learn, statsmodels, and rank-bm25 for retrieval. PDF text extraction was performed using standard open-source tools. Model inference and verification were executed via the OpenAI API with logging enabled to support reproducibility. Detailed package versions, runtime configurations, and execution scripts are documented in the analysis code repository [30], which may be accessed as described in the “Data Availability” statement.

### Evaluation Framework and Analysis Rationale

The RtV procedure operationalizes hallucination assessment by explicitly separating information retrieval from claim verification. This design enables a distinction between failures of evidence access and failures of model reasoning. The resulting evidence support and hallucination metrics complement conventional accuracy and agreement measures by directly assessing whether AI-generated medical information is grounded in source documents. This evaluation framework supports reproducible auditing, cross-model comparison, and systematic analysis of hallucination risk in AI-generated medical information for digital health systems.

### Ethical Considerations

#### Human Research Determination

This study did not meet the regulatory definition of research involving human participants and was therefore not submitted to an institutional review board for review or an exemption determination. Under the US Federal Policy for the Protection of Human Subjects, a human participant is a living individual about whom an investigator obtains information or biospecimens through intervention or interaction with the individual, or obtains, uses, studies, analyzes, or generates identifiable private information or identifiable biospecimens (45 CFR 46.102[e]) [31]. The materials analyzed in this study were exclusively previously published, publicly disseminated documents: full-text reports of randomized controlled trials and the structured RoB annotations published as part of a 2023 Cochrane systematic review [19]. The investigators had no intervention or interaction with any living individual and did not obtain, use, analyze, or generate any identifiable private information or identifiable biospecimens. The unit of analysis was the published trial report, not the trial participant.

The determination that this activity does not constitute human participants research was made by the investigators in accordance with the policies of the Human Research Protection Program of the National Institutes of Health Intramural Research Program, which establish the responsibilities and requirements for institutional review board review of human research conducted by the National Institutes of Health Intramural Research Program [32,33]. As no application for ethics review was submitted, no case, protocol, or application number was assigned to this study.

#### Ethics Approval of the Original Trials

Ethics approval and participant consent for each of the 97 included randomized controlled trials were obtained by the investigators of those trials and are documented in the respective primary publications; approval status is summarized in the source Cochrane review [19]. Our analysis neither extended nor modified the original data collection nor made any attempt to reidentify participants.

#### Informed Consent

No informed consent was sought or required for this analysis, as no human participants were enrolled and no individual-level participant data were accessed. Only aggregate results reported in the published trial documents were used.

#### Privacy and Confidentiality

No protected health information or individually identifiable participant data were accessed, stored, or transmitted at any stage of this study. The text and tables submitted to commercial LLM application programming interfaces consisted solely of published trial-report content and contained no personal or identifiable information. No identifiable data appear in the manuscript, tables, figures, multimedia appendices, or released analysis code.

#### Permission to Use Data and Participant Compensation

The Cochrane systematic review and the included trial reports are third-party copyrighted materials that were accessed under institutional subscriptions and licenses held by the National Institutes of Health Library. These materials were used solely for the methodological research described here and were not redistributed; readers should obtain them directly from the Cochrane Library or the original publishers. No study participants were involved in this research, and no compensation was provided.

## Results

### Accuracy Relative to Human RoB Assessment

The RoB 2 tool structures the assessment of bias across 5 methodological domains: domain 1 assesses bias arising from the randomization process; domain 2 evaluates bias due to deviations from intended interventions; domain 3 examines bias due to missing outcome data; domain 4 addresses bias in measurement of the outcome; and domain 5 considers bias in selection of the reported result. These domain-level judgments are integrated into an overall RoB conclusion. Using human RoB 2 assessments as the reference standard, all 3 models demonstrated high binary accuracy across domains, with the strongest performance in domains 1 and 5, where binary accuracy ranged from approximately 93% to 98%. By contrast, exact (ordinal) accuracy was substantially lower across domains, indicating frequent mismatches in severity classification despite broad agreement on overall risk direction.

GPT-5 showed the strongest overall task performance. It achieved perfect performance on the overall RoB conclusion, with accuracy, sensitivity, specificity, *F*_1_-score, and Youden *J* all of 1.00, and attained the highest exact accuracy in most RoB 2 assessment domains, including 0.64 in domain 1, 0.54-0.71 in domains 2 and 3, and 0.42-0.45 in domains 4 and 5. OpenAI o3-mini closely tracked GPT-5 on binary accuracy but consistently showed lower exact accuracy, while GPT-3.5 demonstrated lower performance on both measures across all domains.

Incorporating evidence support revealed a substantial divergence between apparent decision-level correctness and documentary grounding. The accuracy-support gap (binary accuracy minus evidence support) ranged from 11% to 37% across domains and models (Table 1). Notably, large gaps persisted even in domains with high binary accuracy, particularly domains 2 and 5, where gaps exceeded 30% for all models. For the overall RoB conclusion, gaps exceeded 35% across models, indicating that many apparently correct judgments were not explicitly supported by the trial reports.

Together, these findings show that while LLMs often identify the correct direction of risk, they frequently do so without sufficient evidentiary support. The divergence among binary accuracy, exact accuracy, and evidence support highlights a key limitation of agreement-based evaluation and underscores the importance of assessing whether AI-generated judgments are grounded in source documents. Detailed results are presented in Table 1 and Figure 2.

**Table 1:** Performance by model and risk-of-bias domain^a^.

Model and domain		Exact accuracy, %	Accuracy, %	Sensitivity	Specificity	*F*_1_-score	Youden index (*J*)^b^	Accuracy-support gap, %
**GPT-3.5**								
	Domain 1	60.06	97.59	0.99	0.95	0.98	0.95	15.75
	Domain 2	50.97	93.57	0.95	0.90	0.95	0.86	36.99
	Domain 3	66.59	81.33	0.76	0.88	0.81	0.64	25.91
	Domain 4	37.67	78.46	0.61	0.86	0.64	0.47	14.45
	Domain 5	39.63	93.37	0.89	0.95	0.86	0.84	30.42
	Overall risk of bias^c^	74.44	96.77	0.90	1.00	0.95	0.90	35.82
**GPT-5**								
	Domain 1	64.23	97.64	0.99	0.96	0.98	0.95	16.45
	Domain 2	54.22	93.69	0.95	0.92	0.95	0.87	33.92
	Domain 3	71.14	81.25	0.75	0.88	0.81	0.63	23.79
	Domain 4	41.53	79.75	0.61	0.88	0.65	0.49	12.72
	Domain 5	44.92	94.72	0.92	0.95	0.89	0.88	33.19
	Overall risk of bias^c^	90.99	100.00	1.00	1.00	1.00	1.00	37.41
**OpenAI o3-mini**								
	Domain 1	62.40	97.76	0.99	0.96	0.98	0.95	18.50
	Domain 2	49.70	93.48	0.95	0.92	0.95	0.86	37.22
	Domain 3	69.76	82.52	0.77	0.89	0.82	0.66	26.15
	Domain 4	37.60	78.26	0.61	0.86	0.64	0.47	11.28
	Domain 5	39.43	92.99	0.91	0.94	0.86	0.85	29.78
	Overall risk of bias^c^	77.35	100.00	1.00	1.00	1.00	1.00	37.08

^a^Risk-of-bias 2 domains: domain 1, bias arising from the randomization process (evaluates sequence generation and allocation concealment); domain 2, bias due to deviations from intended interventions (assesses effect of assignment or adherence to interventions); domain 3, bias due to missing outcome data (analyzes whether results differ based on missing data); domain 4, bias in measurement of the outcome (checks whether outcome assessment is blinded or affected by measurement methods); and domain 5, bias in selection of the reported result (assesses whether results are reported selectively from analyses).

^b^Calculated as sensitivity + specificity − 1.

^c^Overall risk-of-bias conclusion.

**Figure 2:**
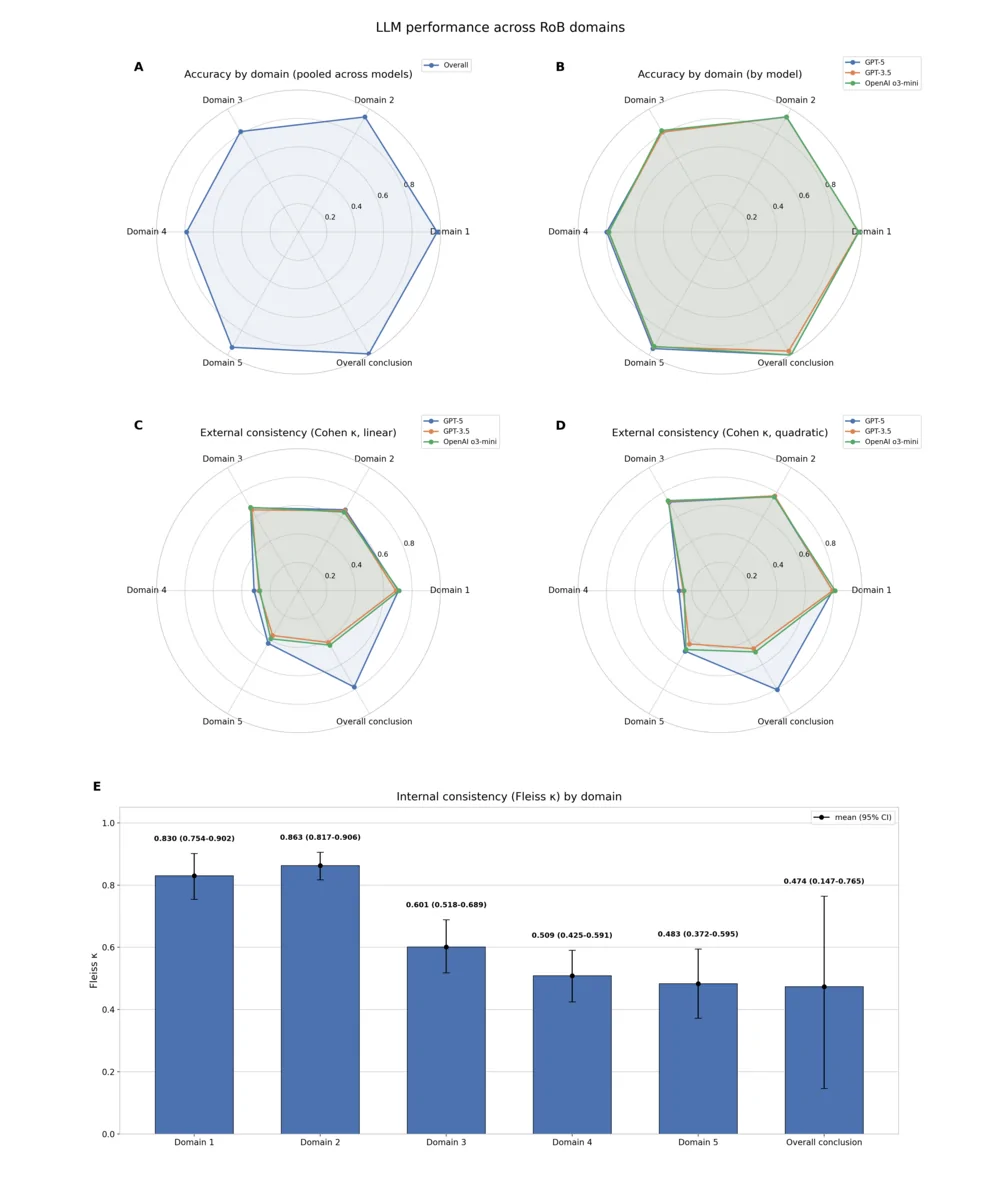
Large language model (LLM) performance across risk of bias (RoB) 2 domains. (A) Accuracy by domain (pooled accross models). (B) Accuracy by domain (by model). (C) External consistency (Cohen κ, linear). (D) External consistency (Cohen κ, quadratic). (E) Internal consistency (Fleiss κ) by domain.

### Agreement and Consistency of RoB Judgments

We evaluated the consistency of LLM outputs along 2 dimensions: external agreement with human RoB assessments and internal agreement across models. External agreement was assessed using Cohen κ, whereas internal agreement was assessed using Fleiss κ.

External agreement with human ratings was substantial in domains 1-3, with linear κ values ranging from approximately 0.64 to 0.71 and quadratic κ values from approximately 0.72 to 0.81. Agreement was lower in domain 4, where κ values were in the fair range (linear κ approximately 0.27-0.31; and quadratic κ approximately 0.25-0.29), and fair to moderate in domain 5 (linear κ approximately 0.36-0.43; and quadratic κ approximately 0.43-0.49). GPT-5 showed notably higher agreement on the overall RoB conclusion (linear κ=0.78; and quadratic κ=0.81) than OpenAI o3-mini and GPT-3.5 (linear κ approximately 0.42-0.44; and quadratic κ approximately 0.47-0.50).

Across domains, quadratic κ values consistently exceeded linear κ values, indicating that most disagreements involved adjacent categories on the ordinal RoB 2 scale rather than large category gaps. Internal consistency across models was strong in domain 1 (Fleiss κ=0.83) and domain 2 (Fleiss κ=0.86), and moderate in domains 3-5 (Fleiss κ of approximately 0.49-0.60) and for the overall RoB conclusion (Fleiss κ=0.51). This pattern suggests high convergence in domains governed by explicit methodological criteria and greater divergence in domains requiring more nuanced interpretation.

Detailed agreement results are reported in Table 2. Figure 2 illustrates LLM performance across RoB 2 domains: Figure 2A-2D presents binary accuracy and external consistency (Cohen κ with linear and quadratic weighting) across domains using radar plots, and Figure 2E shows internal consistency across the 3 LLMs, measured by Fleiss κ for each RoB 2 domain, with 95% CIs.

**Table 2:** Large language model response agreement analysis.

Model and domain^a^		Cohen κ linear^b^	Cohen κ quadratic^b^	Fleiss κ^b^
**GPT-3.5**				
	Domain 1	0.69	0.79	0.83
	Domain 2	0.65	0.77	0.86
	Domain 3	0.66	0.73	0.60
	Domain 4	0.28	0.26	0.51
	Domain 5	0.36	0.43	0.49
	Overall risk of bias^c^	0.42	0.47	0.51
**GPT-5**				
	Domain 1	0.70	0.79	0.83
	Domain 2	0.66	0.77	0.86
	Domain 3	0.67	0.72	0.60
	Domain 4	0.31	0.29	0.51
	Domain 5	0.43	0.49	0.49
	Overall risk of bias^c^	0.78	0.81	0.51
**OpenAI o3-mini**				
	Domain 1	0.71	0.81	0.83
	Domain 2	0.64	0.76	0.86
	Domain 3	0.68	0.73	0.60
	Domain 4	0.27	0.25	0.51
	Domain 5	0.39	0.48	0.49
	Overall risk of bias^c^	0.44	0.50	0.51

^a^Risk-of-bias 2 domains: domain 1, bias arising from the randomization process (evaluates sequence generation and allocation concealment); domain 2, bias due to deviations from intended interventions (assesses effect of assignment or adherence to interventions); domain 3, bias due to missing outcome data (analyzes whether results differ based on missing data); domain 4, bias in measurement of the outcome (checks whether outcome assessment is blinded or affected by measurement methods); and domain 5, bias in selection of the reported result (assesses whether results are reported selectively from analyses).

^b^Cohen κ linear and Cohen κ quadratic denote the weighting schemes for ordinal categories; quadratic penalizes larger category gaps more strongly and typically yields higher κ when disagreements are near-adjacent. Fleiss κ quantifies intermodel agreement among the 3 large language models.

^c^Overall risk-of-bias conclusion.

### Evidence Support and Hallucination

To complement accuracy and agreement analyses, we evaluated evidence support and hallucination using the RtV metrics. As shown in Figure 3A, overall evidence support rates were approximately 60%-65% across models, whereas both conservative and strict hallucination rates ranged from approximately 35% to 37%. These findings indicate that a substantial proportion of AI-generated judgments lacked explicit documentary support, even when they aligned with human assessments at the decision level.

**Figure 3:**
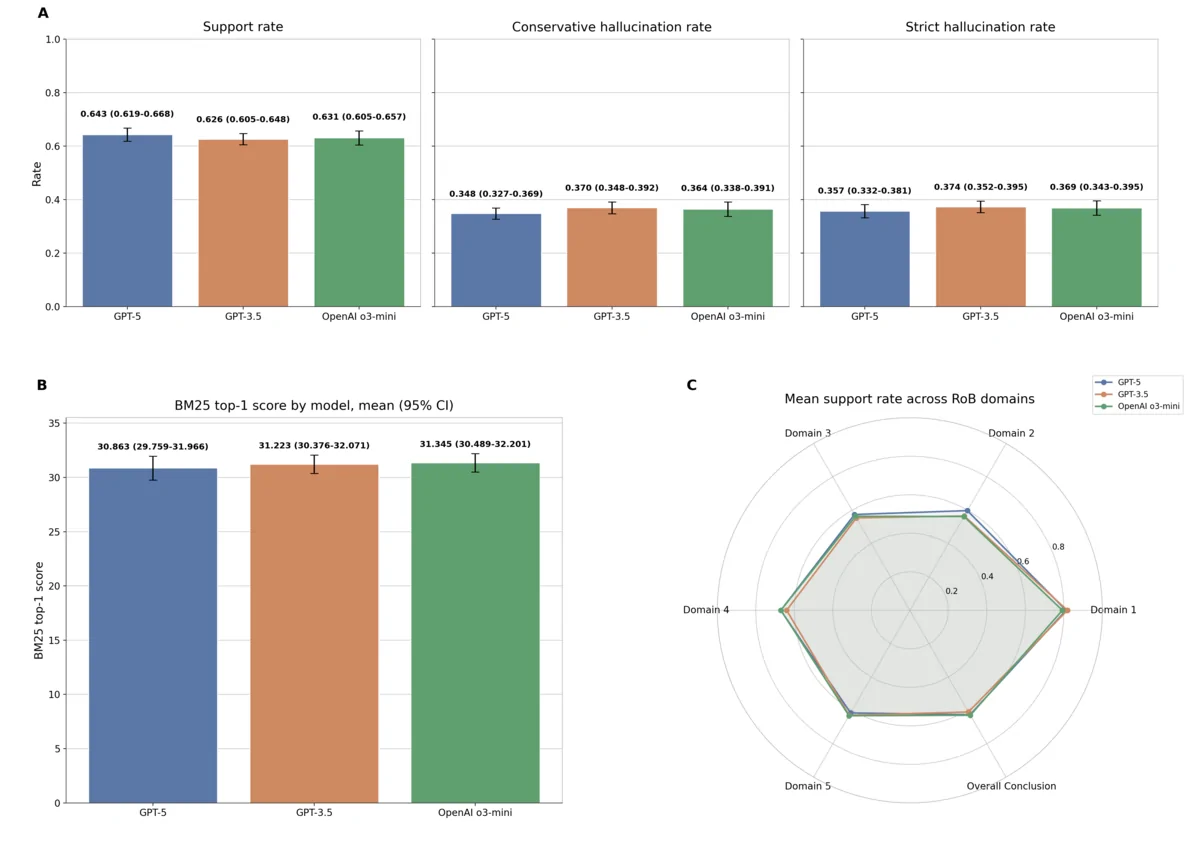
Evidence support, hallucination rates, and retrieval diagnostics across models. (A) Support rate; Conservative hallucination rate; Strict hallucination rate. (B) BM25 top-1 score by model, mean (95% CI). (C) Mean support rate accross RoB domains. CI: confidence interval; BM25: Best Matching 25; RoB: risk of bias.

GPT-5 achieved the highest mean evidence support rate (mean 64.3%, SD 5.9%) and the lowest hallucination rates (conservative: mean 34.8%, SD 5.1%; and strict: mean 35.7%, SD 5.9%), closely followed by OpenAI o3-mini. GPT-3.5 showed modestly lower evidence support and higher hallucination rates across all 3 measures. Topic (outcome)-specific results in Table 3 were consistent with these aggregate findings. For outcomes related to distress or posttraumatic stress disorder, GPT-5 achieved 69.04% evidence support with a strict hallucination rate of 30.96%, whereas GPT-3.5 showed lower support for anxiety symptoms and social functioning outcomes. OpenAI o3-mini performed competitively across most domains and achieved the highest support for adverse events (76.79%); however, this estimate was based on a single study and should therefore be interpreted with caution.

Retrieval diagnostics suggested that differences in evidence support were not primarily driven by differences in retrieval strength. Mean top-1 BM25 scores (Figure 3B) clustered near 31 across GPT-5, OpenAI o3-mini, and GPT-3.5, with overlapping 95% CIs. As BM25 scores reflect lexical relevance between queries and retrieved passages, these similar values suggest comparable access to potentially relevant evidence across models. The observed differences in evidence support therefore likely reflect downstream reasoning and claim formulation rather than systematic differences in retrieval strength. One exception was observed for GPT-5 in the adverse events domain, where a lower mean BM25 score coincided with reduced evidence support, suggesting possible underretrieval or weaker alignment between retrieved passages and generated claims in this domain. This finding highlights the sensitivity of evidence grounding to retrieval quality when relevant signals are sparse or heterogeneously reported.

To complement BM25 lexical retrieval, we computed embedding-based semantic similarity between the LLM’s stated rationale and the retrieved passages as an additional grounding diagnostic (Multimedia Appendix 3). Semantic similarity scores were uniformly low across all models and topics. To distinguish surface-language divergence from potential retrieval failure, we stratified semantic similarity by verification verdict (Multimedia Appendix 4). Mean similarity decreased monotonically from supported to contradicted, not found, and out-of-scope verdicts, but the difference between supported and nonsupported items was small (max top-*k*: Mann-Whitney *z*=20.14, *P*<.001, rank-biserial *r*=0.17; medians 0.49 vs 0.46). Critically, even items with confirmed documentary support showed low absolute similarity (max top-*k*≈0.49; mean top-*k*≈0.42), indicating that semantic similarity was not sufficient to distinguish supported from unsupported items. Semantic similarity also correlated only weakly with the BM25 top-1 score (Spearman ρ=0.22, *P*<.001), indicating that lexical and embedding metrics capture complementary rather than redundant dimensions of grounding. The pervasively low similarity is consistent with systematic divergence between the model’s rationale phrasing and the source text, while the modest verdict gradient suggests that weaker semantic grounding may contribute secondarily, particularly among not found and out-of-scope items. We acknowledge that, for a subset of unsupported verdicts, low similarity may partly reflect genuine underretrieval rather than paraphrasing alone, and these explanations cannot be fully disentangled using lexical and embedding diagnostics alone.

Domain-level patterns further indicated that task structure influenced evidence grounding. Radar plots of mean support rate across RoB 2 domains (Figure 3C) showed higher evidence support in domain 1 (approximately 0.78-0.80) and lower support in domain 3 (approximately 0.56-0.59) across models, potentially reflecting differences in reporting clarity and methodological complexity. GPT-5 showed modestly higher support in domains 2 and 3, whereas OpenAI o3-mini performed slightly better in domain 4; overall differences across models were small.

To assess the robustness of the RtV method to retrieval depth, we conducted a sensitivity analysis varying the number of BM25-retrieved passages provided to the verdict LLM (k=1, 3, 5). As shown in Figure 4, evidence support rates increased substantially and consistently with larger k across all 8 topics, while conservative and strict hallucination rates decreased correspondingly. This pattern was consistent across topics, indicating that richer retrieval context substantially increased the availability of documentary support for generated claims. Conversely, restricting context to a single passage (k=1) resulted in markedly elevated hallucination rates, reflecting the difficulty of grounding judgments using minimal evidence.

To further examine the role of the verdict LLM in evidence evaluation, we compared outcomes produced by a judge model without explicit reasoning capability (GPT-3.5-turbo) with those produced by a reasoning-capable judge (GPT-5), as shown in Figure 5. The reasoning-capable judge assigned higher evidence support rates across most topics and produced more decisive conservative hallucination classifications. By contrast, the judge without reasoning classified a substantially larger proportion of items as “out of scope” rather than resolving them into supported or contradicted verdicts, resulting in higher strict hallucination rates overall. These findings suggest that judge model reasoning capacity influences verdict discrimination in the RtV framework: reasoning-capable models may be better able to interpret ambiguous retrieval contexts and render substantive evidence judgments, whereas models without explicit reasoning may be more likely to abstain when evidence is indirect or incomplete. This has practical implications for RtV pipeline design, suggesting that verdict reliability may be sensitive to the reasoning depth of the judge LLM, in addition to retrieval quality.

The comparison in Figure 5 isolates the effect of reasoning capacity in the verifier role. It does not address how the reasoning depth of the generator model affects the model’s own tendency to produce unsupported claims. To examine this separate question, we regenerated all RoB assessments with GPT-5 at minimal reasoning and evaluated them using the identical RtV pipeline. Lower reasoning depth in the generator model reduced both evidence support (65.5% to 55.9% pooled) and binary accuracy (89.5% to 77.7% pooled), without a comparable change in retrieval scores. Full results are reported in Multimedia Appendix 8.

In summary, GPT-5 offered the most favorable balance between evidence support and hallucination control, with OpenAI o3-mini remaining closely comparable and GPT-3.5 exhibiting the highest tendency toward unsupported claims. The consistency of BM25 retrieval scores across models suggests that differences in hallucination rates were not primarily attributable to differences in retrieval strength and may instead reflect differences in reasoning and interpretation. The sensitivity analyses further demonstrated that both retrieval depth and judge model reasoning capacity are important determinants of RtV evaluation outcomes, and that an appropriate top-k configuration combined with a reasoning-capable judge may provide a robust design choice for this framework. Detailed per-study forest plots of evidence support and hallucination rates are provided in Multimedia Appendices 9-11.

**Table 3:** Evidence support and hallucination rates of AI-generated medical information across outcome domains.

Model and topic		Support rate (%), mean (SD)	Conservative hallucination rate (%), mean (SD)	Strict hallucination rate (%), mean (SD)	Mean BM25^a^ score^b^, mean (SD)	Semantic similarity^c^, mean (SD)
**GPT-3.5**						
	Adverse events	64.29 (0.00)	35.71 (0.00)	35.71 (0.00)	28.80 (0.00)	0.37 (0.00)
	Anxiety symptoms	57.76 (4.96)	42.12 (5.12)	42.24 (4.96)	26.32 (0.86)	0.34 (0.01)
	Depressive symptoms	61.56 (4.34)	37.93 (4.50)	38.44 (4.34)	25.61 (0.25)	0.34 (0.01)
	Diagnosis of mental disorders	63.10 (5.38)	36.61 (5.85)	36.90 (5.38)	26.60 (1.04)	0.34 (0.01)
	Distress/posttraumatic stress disorder symptoms	64.25 (1.90)	34.72 (1.44)	35.75 (1.90)	24.73 (1.15)	0.35 (0.01)
	Psychological functioning and impairment	65.67 (6.37)	34.33 (6.37)	34.33 (6.37)	24.46 (3.82)	0.35 (0.01)
	Quality of life	67.27 (0.87)	32.23 (0.67)	32.73 (0.87)	27.96 (0.84)	0.37 (0.02)
	Social outcomes	58.31 (6.43)	41.39 (6.57)	41.69 (6.43)	26.95 (0.13)	0.34 (0.01)
**GPT-5**						
	Adverse events	50.00 (0.00)	41.07 (0.00)	50.00 (0.00)	19.59 (0.00)	0.40 (0.00)
	Anxiety symptoms	62.33 (1.61)	37.05 (1.22)	37.67 (1.61)	26.39 (1.49)	0.34 (0.01)
	Depressive symptoms	61.61 (6.12)	37.77 (5.89)	38.39 (6.12)	25.50 (0.37)	0.35 (0.01)
	Diagnosis of mental disorders	67.11 (6.26)	32.00 (5.36)	32.89 (6.26)	26.02 (0.51)	0.33 (0.01)
	Distress/posttraumatic stress disorder symptoms	69.04 (2.26)	30.79 (1.97)	30.96 (2.26)	24.33 (1.28)	0.34 (0.00)
	Psychological functioning and impairment	64.29 (3.58)	35.25 (3.18)	35.71 (3.58)	25.17 (2.74)	0.35 (0.01)
	Quality of life	66.46 (9.86)	33.09 (10.27)	33.54 (9.86)	28.00 (1.21)	0.37 (0.01)
	Social outcomes	64.21 (1.20)	35.71 (1.19)	35.79 (1.20)	27.97 (1.44)	0.36 (0.00)
**OpenAI o3-mini**						
	Adverse events	76.79 (0.00)	21.43 (0.00)	23.21 (0.00)	26.97 (0.00)	0.36 (0.00)
	Anxiety symptoms	57.60 (3.93)	42.17 (4.12)	42.40 (3.93)	26.29 (0.33)	0.34 (0.01)
	Depressive symptoms	62.03 (2.92)	37.62 (2.82)	37.97 (2.92)	25.70 (0.24)	0.35 (0.01)
	Diagnosis of mental disorders	67.56 (4.58)	32.29 (4.78)	32.44 (4.58)	26.01 (0.67)	0.33 (0.02)
	Distress/posttraumatic stress disorder symptoms	64.79 (1.67)	35.07 (1.60)	35.21 (1.67)	25.07 (1.29)	0.35 (0.00)
	Psychological functioning and impairment	62.70 (1.38)	37.04 (1.22)	37.30 (1.38)	24.83 (3.78)	0.35 (0.00)
	Quality of life	63.18 (11.67)	36.30 (12.20)	36.82 (11.67)	27.50 (0.55)	0.37 (0.01)
	Social outcomes	59.23 (5.81)	39.64 (4.89)	40.77 (5.81)	28.36 (1.69)	0.36 (0.02)

^a^BM25: Best Matching 25.

^b^Mean BM25 score is the mean retrieval similarity score of the passages retrieved from the original clinical reports.

^c^Semantic similarity is the mean cosine similarity between the large language model’s explanation text and the BM25-retrieved passages, computed using the sentence-transformers/all-MiniLM-L6-v2 encoder with L2-normalized embeddings, and is reported as a grounding diagnostic alongside BM25 (see Multimedia Appendix 4).

**Figure 4:**
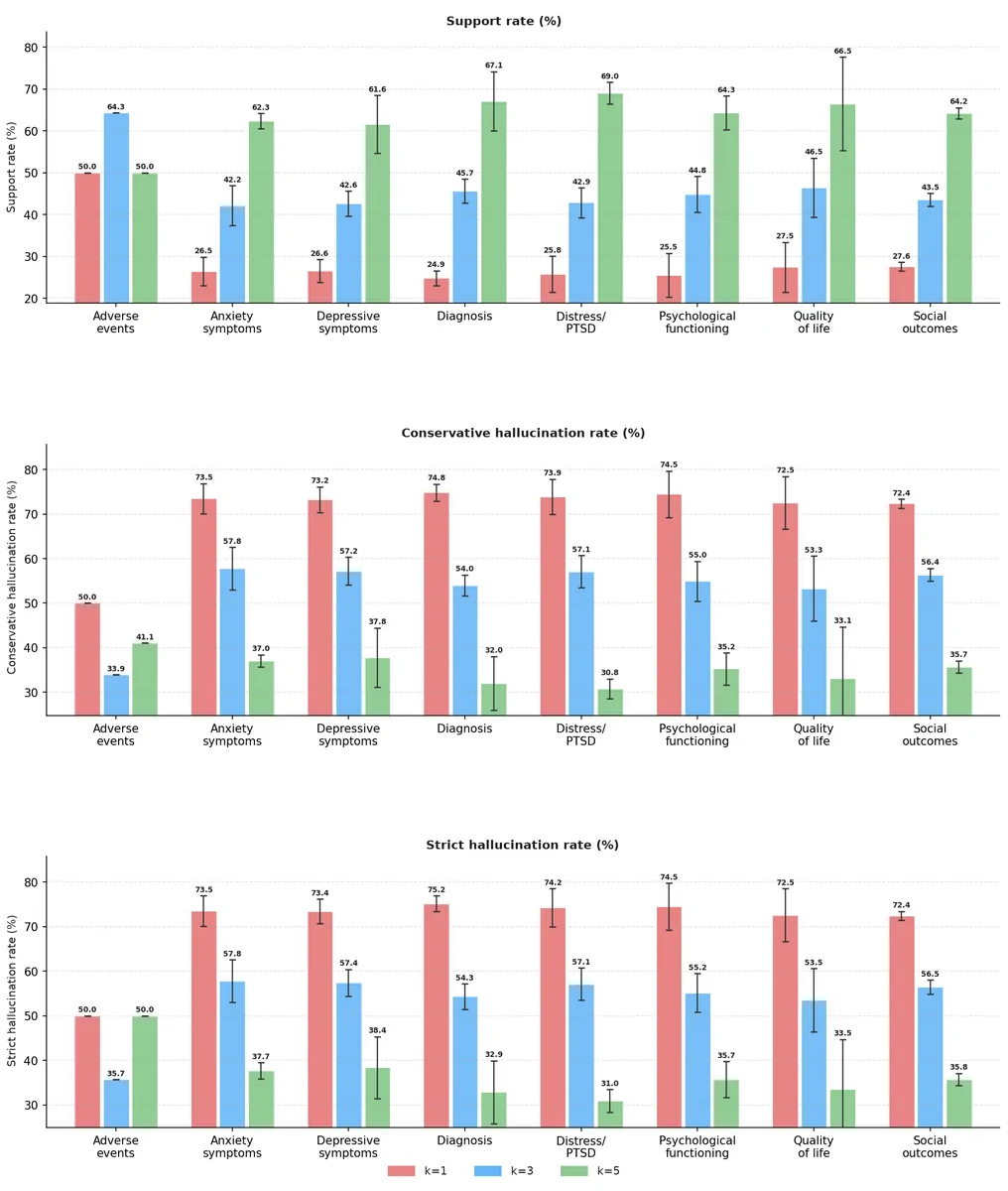
Best Matching 25 top-k retrieval sensitivity: evidence support and hallucination rates across topics (k=1, 3, 5). PTSD: posttraumatic stress disorder.

**Figure 5:**
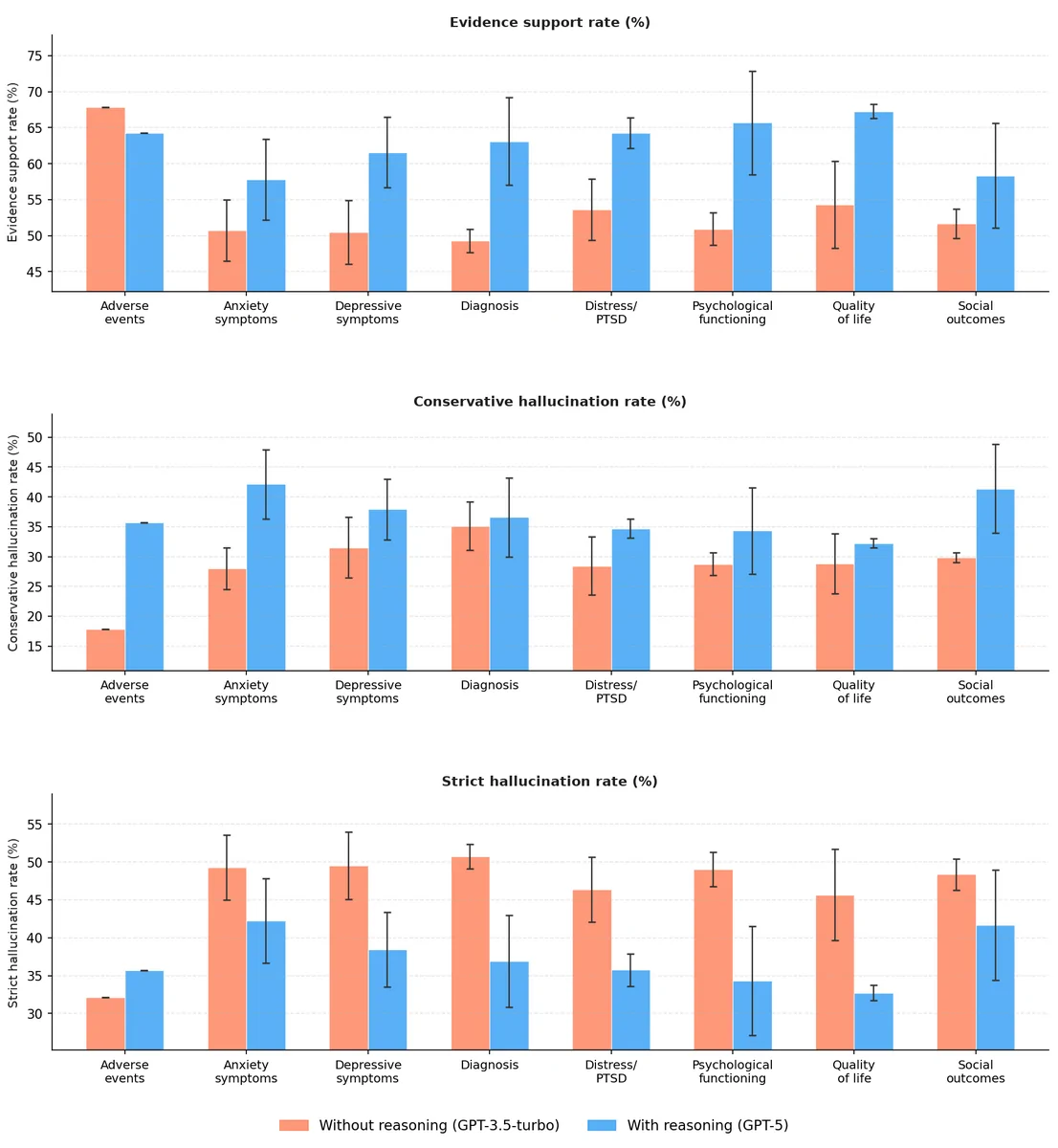
Effect of judge model reasoning on evidence evaluation outcomes: without reasoning (GPT-3.5-turbo) versus with reasoning (GPT-5). PTSD: posttraumatic stress disorder.

## Discussion

### Principal Findings

This study introduced an RtV framework to measure evidence support and hallucination in AI-generated medical information at the claim level, using RoB assessment as a concrete and well-characterized use case. By explicitly separating information retrieval from claim verification, the new method evaluates whether AI-generated statements are grounded in source documents rather than unsupported by the available evidence, offering a distinction that conventional accuracy and agreement metrics do not capture.

The principal finding is that high decision-level accuracy and strong agreement with human judgments do not guarantee evidence grounding. Binary accuracy of AI-generated RoB judgments was high across all domains (90%-98%), whereas evidence support rates clustered at only 60%-65%. Furthermore, conservative hallucination rates remained substantial at approximately 35%-37%, and exact accuracy was substantially lower than binary accuracy (42%-71%). These results reflect substantial classification disagreements despite correct directional judgments. GPT-5 achieved the most favorable balance between accuracy and evidence grounding, with the highest mean evidence support rate of 64.3% and the lowest strict hallucination rate of 35.7%. OpenAI o3-mini performed comparably, while GPT-3.5 exhibited higher rates of unsupported claims. Retrieval diagnostics showed that mean top-1 BM25 scores were similar across models (approximately 30-31). This suggests that differences in hallucination behavior were not primarily attributable to differences in retrieval strength.

### Interpretation, Implications, and Comparison With Prior Work

#### Evidence Grounding as a Distinct Assessment

A crucial finding of this study is that accuracy and evidence support are empirically dissociable. AI-generated RoB judgments frequently agreed with human judgments while simultaneously lacking evidence grounding in the original trial reports. This inconsistency reflects a known and well-documented limitation of LLMs: their tendency to generate fluent, plausible, and contextually appropriate outputs that nonetheless do not correspond to information explicitly present in source materials. Prior work has found hallucinations in several relevant domains, including fabricated or inaccurate references in systematic reviews [7,9], unsupported claims in clinical text summarization [8], and adversarial exploitation of false clinical details in decision support systems [11]. The RtV framework extends these findings by quantifying hallucination as a measurable, claim-level property. Thus, it enables quantitative comparison across models, domains, and tasks rather than relying on expert impression or post hoc review.

#### Relationship to Prior Evaluations of LLMs for Evidence Appraisal

Recent studies have examined the ability of LLMs to assist with RoB assessment and have reported promising agreement with human reviewers [18,24-27]. For example, Lai et al [18,27] demonstrated that LLMs can perform RoB assessments at a level comparable to trained reviewers in structured appraisal tasks, and Rose et al [25,26] found moderate to substantial interrater agreement between ChatGPT-4o and human reviewers across Cochrane RoB review domains. AI, or machine-assisted, RoB assessment has also been explored as a means to improve scalability in evidence synthesis workflows [24]. However, these studies mainly focus on decision-level agreement and do not assess whether model outputs are grounded in the trial reports used as source evidence. Our study addresses this gap by demonstrating that agreement and evidence grounding capture different aspects of model reliability, and that high agreement does not imply that generated claims are traceable to source documents. This distinction is especially consequential in evidence-based medicine, where the integrity of appraisal decisions depends not only on reaching the correct conclusion but also on anchoring that conclusion in explicit, documented evidence.

#### Implication for Digital Health System and Evaluation

From an application perspective, the RtV framework is feasible for routine integration into evidence-focused workflows. It required end-to-end runtimes of 50-158 seconds per study, depending on model choice, and the computational requirements were modest. The retrieval component uses sparse lexical indexing with BM25, and verification requires a single constrained model (online or locally deployed) call per claim. The framework also supports human-in-the-loop workflows, where unsupported or contradicted claims can be selectively escalated for expert review, while supported claims may be accepted with reduced oversight. This selective verification approach aligns with real-world clinical and informatics workflows by preserving human expertise for cases where it is most needed. Effective use of the RtV framework requires familiarity with systematic review methodology and the Cochrane RoB 2 instrument. Users adjudicating escalated claims should have training in clinical epidemiology or evidence synthesis; deployment in fully automated settings without expert oversight is not recommended at the current stage of validation.

### Limitations

A limitation of this study is that the retrieval component relied on BM25 lexical matching, which may miss paraphrased, implicitly stated, or semantically equivalent evidence not captured by term overlap. Although the RtV framework shares structural resemblance to retrieval-augmented generation pipelines, it serves an evaluative rather than generative purpose: retrieval locates candidate evidence passages for post hoc claim verification rather than supplying context to guide response generation. Prebuilt retrieval-augmented generation pipelines are therefore not directly applicable to this evaluative role. However, dense retrieval methods, including embedding-based approaches, are architecturally compatible with the RtV framework and represent a concrete direction for future work. The addition of embedding-based semantic similarity scores in this study already moves in this direction by providing a dense grounding diagnostic alongside BM25, while keeping final verdicts anchored to the structured verification step. PDF extraction quality, document chunking strategy, and top-k passage selection may further affect retrieval performance in ways not fully characterized here.

Verification relied on a single LLM judge, which may introduce systematic bias in borderline or ambiguous cases; multijudge adjudication or ensemble verification approaches could reduce this risk, albeit at the cost of increased runtime. The empirical evaluation was conducted using a single Cochrane systematic review on mental health and well-being interventions, comprising 97 randomized controlled trials. Generalizability to other clinical specialties, review types, or evidence appraisal frameworks has not been established and represents an important direction for future work. Additionally, RoB assessment is inherently subject to variable reporting quality and interreviewer subjectivity in the source trials, which may contribute to hallucination rates independently of model behavior.

A related question concerns the reasoning depth of the generator model itself. A supplementary analysis (Multimedia Appendix 8) examined this by regenerating assessments with GPT-5 at minimal reasoning and found that lower reasoning depth reduced both evidence grounding and task accuracy. A full characterization across multiple reasoning levels, models, and clinical domains was beyond the scope of this study and remains an important direction for future work.

The included RCTs were drawn from a Cochrane review focused on low- and middle-income country settings, representing a geographically and demographically specific evidence base. Demographic harmonization across trials was not performed, and the extent to which findings generalize to evidence appraisal tasks in high-income settings, other clinical specialties, or other evidence appraisal frameworks remains to be established. In deployment settings, input document quality should be assessed before RtV processing. Studies with poor-quality PDFs, including scanned images without optical character recognition, low-resolution text extraction, or incomplete full-text availability, may yield elevated hallucination rates because of degraded retrieval and should be flagged or excluded before pipeline application. These limitations are addressable through hybrid retrieval combining sparse and dense methods, improved document parsing, uncertainty-aware verification, multijudge adjudication, and cross-domain validation. All proposed extensions are compatible with the RtV framework and represent priorities for future work.

### Conclusions

This study reveals that AI-generated medical information can achieve high decision-level accuracy while a substantial proportion of outputs remain unsupported by the source documents underlying the judgments. By reframing hallucination as a problem of evidence grounding rather than disagreement with human judgments, the RtV framework provides a reproducible, source-linked evaluation paradigm that captures a clinically and informatically important dimension of model reliability that is not captured by conventional accuracy and agreement metrics.

The broader implication is that as LLMs are increasingly integrated into clinical decision support, evidence synthesis, and guideline development workflows, evaluation standards must evolve to require explicit evidence traceability alongside task accuracy. Regulatory and institutional governance frameworks for AI in medicine will need to address not only whether models reach correct conclusions but also whether those conclusions can be transparently linked to authoritative source materials. The RtV approach offers a technically feasible and workflow-compatible mechanism for operationalizing this requirement. Wider adoption of evidence-grounding metrics in model development, benchmarking, procurement, and postdeployment monitoring will be essential for promoting transparency, accountability, and the safe and trustworthy integration of AI into evidence-based medicine and medical informatics.

## Appendix

Multimedia Appendix 1 Prompt engineering with context for structured RoB 2 response. RoB: risk of bias.

Multimedia Appendix 2 RoB 2 prompt and response example. RoB: risk of bias

Multimedia Appendix 3 Algorithm for embedding-based semantic grounding for hallucination assessment.

Multimedia Appendix 4 Analysis of semantic similarity by verification verdict and its relationship to retrieval quality.

Multimedia Appendix 5 Algorithm for quantitatively measuring LLM responses using BM25 retrieval and LLM verdicts. LLM: large language model.

Multimedia Appendix 6 An example retrieve-then-verify prompt and verification response.

Multimedia Appendix 7 An example of retrieve-then-verify verification rationale (BM25 retrieval scores, retrieved passages, and verdict justification).

Multimedia Appendix 8 Effect of generator-model reasoning depth on evidence support and hallucination (GPT-5 minimal vs medium reasoning).

Multimedia Appendix 9 Support rate by study (reverse order).

Multimedia Appendix 10 Strict hallucination rate by study (reverse order).

Multimedia Appendix 11 Conservative hallucination rate by study (reverse order).

## Abbreviations

BM25: Best Matching 25
LLM: large language model
RoB: risk of bias
RtV: retrieve-then-verify

## Data Availability

The review data are available from the Cochrane Library. The Python code for RoB prompting, BM25 retrieval, retrieve-then-verify evaluation, and analyses is not publicly available because the repository also contains code under continued development for related ongoing work. However, the code is available from the corresponding author (SA) upon reasonable request from academic, noncommercial researchers. No protected health information is included. Original trial PDFs and human risk-of-bias annotations are subject to third-party licensing and are not redistributed; users should obtain these materials directly from the Cochrane Library or the original publishers.
