# Supplementary material for: Retrieve-Then-Verify for Evaluating Evidence Support and Hallucination in Large Language Model–Generated Medical Information: Empirical Study
Source: JMIR AI. 2026 Sep 16;5:e93761. doi: 10.2196/93761 (PMC13628037; doi:10.2196/93761)
Supplement: Supplementary file 1 [file ai_v5i1e93761_app1.pdf]

Algorithm Prompt Engineering with Context for Structured RoB 2 Response

Require: CSV file containing relevant studies, with `Study id`, `PDF file path`, `Review Topic`; output path D; LLM m; client connected to OpenAI; rigor criteria text c.
Ensure: One JSON per study at D/`studyName`/`studyName`.$json$.

Read CSV file $\mathcal{S}$.
$p \leftarrow$ first non-empty "review topic" in $\mathcal{S}$; if none, use provided topic.
for each row (`Study` $= s$, `path` $= \pi$) in $\mathcal{S}$ **do**
    $P \leftarrow D/s$; $f_{\text{out}} \leftarrow P/\,(s+$".json")
    if f_{out} exists **then continue** ▷ skip completed studies
    end if
    $x \leftarrow$ ExtractPDFText(π) ▷ join page texts via PDF parser
    $y \leftarrow$ get_rob_response(p, c, x, client, m) ▷ p: review topic; c: RoB 2 criteria text; x: full paper text (extracted from PDF); client: OpenAI client; m: LLM name
    try parse y as JSON; **catch** → wrap as {`"raw_text":` y}
    Create folder P if needed; write JSON to f_{out}
end for
