# Supplementary material for: Retrieve-Then-Verify for Evaluating Evidence Support and Hallucination in Large Language Model–Generated Medical Information: Empirical Study
Source: JMIR AI. 2026 Sep 16;5:e93761. doi: 10.2196/93761 (PMC13628037; doi:10.2196/93761)

Strict hallucination rate by study (reversed order)
(points = study mean; bars = 95% CI; top row = mean across studies)

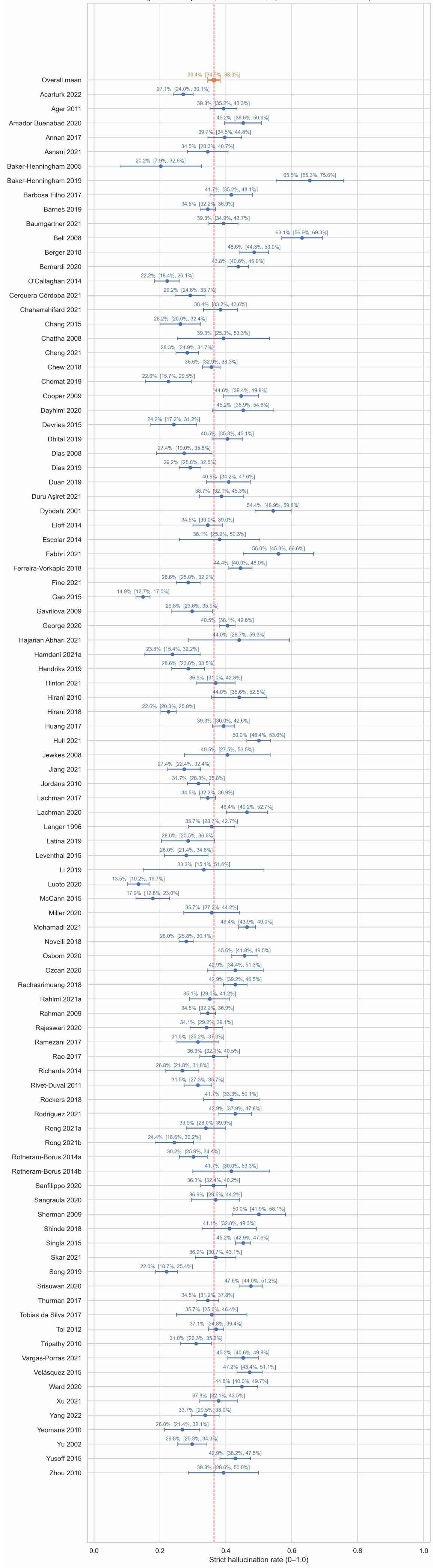

Supplement: Supplementary file 2 [file ai_v5i1e93761_app10.pdf]
