# Supplementary material for: Retrieve-Then-Verify for Evaluating Evidence Support and Hallucination in Large Language Model–Generated Medical Information: Empirical Study
Source: JMIR AI. 2026 Sep 16;5:e93761. doi: 10.2196/93761 (PMC13628037; doi:10.2196/93761)
Supplement: Supplementary file 4 [file ai_v5i1e93761_app2.pdf]

Task and role instruction prompt:

You are a literature reviewer. … Respond to each question using the allowed labels and
provide both a text response and a numerical value:
{ "Yes": 5, "Probably Yes": 4, "Probably": 3, "No": 2, "No information": 1, "Not Applicable": 0 }
{ "High risk": 3, "Some concerns": 2, "Low risk": 1 }
Return valid JSON only in the specified schema. ……

Topic prompt:

the topic to be analyzed: Psychological functioning and impairment at 0-1 months

Context prompt:

the paper to be analyzed: Methods: Effectiveness of a WHO self-help psychological intervention for\npreventing mental disorders among Syrian refugees in Turkey:\na randomized controlled trial\ …… Eligible participants were adult Syrian\nrefugees experiencing psychological distress (General Health Questionnaire ≥3), but without a diagnosis of mental disorder. They were randomly\nassigned either to the SelfHelp Plus arm (consisting of SelfHelp Plus combined with Enhanced Care as Usual, ECAU) or to ECAU only in a 1:1 ratio. ……

RoB 2 prompt:

list of questions to response:
Domain 1: Risk of bias arising from the randomization process
1.1: Was the allocation sequence random?
– Yes / Probably Yes / Probably / No / No information
Instruction: Give comments on your answer.
… …
Domain 1 Conclusion:
– Low risk / High risk / Some concerns
Instruction: Give comments on your answer.
…… (Domains 2–5 and Overall risk of bias) ……

Rigor requirement prompt:

Requirements for rigor in the evaluation criteria throughout the review: Adopt an extraordinarily exacting standard … base your assessment exclusively on the responses … deliver a concise summary. ……

Output instruction prompt:

output instruction: Return valid JSON parsable by json.loads(), no extra text. Each entry must include:

```
{
  "question_id": "...", "response": "...", "numerical": ..., "comment": "...", "reasoning": "..."
}
```

Expected LLM response:

```
[
  {
    "question_id": "1.1",
    "response": "Yes",
    "numerical": 5,
    "comment": "The allocation sequence was clearly described as random, using specialized software and variable block randomization.",
    "reasoning": "The study mentions that participants were randomly assigned using Castor EDC with variable block randomization, supporting a fully random allocation process. "
  },
  {
    "question_id": "1.2",
    "response": "Yes",
    "numerical": 5,
    "comment": "Allocation concealment was ensured as the research team did not have access to the randomization list..",
    "reasoning": "The use of a web-based software that concealed the block size and randomization list ensured that allocation was concealed until participants were enrolled. "
  },
  …… response to each item in the RoB 2 tool …..…
  {
    "question_id": "Overall risk of bias",
    “response": "Some concerns”,
    “numerical”: “2”,
    “comment”: "Overall, the study demonstrates low risk in most domains, with only moderate concerns regarding missing outcome data.",
    “reasoning”: “While domains related to randomization, deviations, measurement, and reporting are robust, the moderate level of missing data introduces some concerns.”
  },
]
```
