# Supplementary material for: Retrieve-Then-Verify for Evaluating Evidence Support and Hallucination in Large Language Model–Generated Medical Information: Empirical Study
Source: JMIR AI. 2026 Sep 16;5:e93761. doi: 10.2196/93761 (PMC13628037; doi:10.2196/93761)
Supplement: Supplementary file 5 [file ai_v5i1e93761_app3.pdf]

Algorithm: Embedding-Based Semantic Grounding for Hallucination Assessment

Require: Hallucination JSON files $\mathcal{J}$; sentence encoder E (`all-MiniLM-L6-v2`); device $d \in \{\text{cpu}, \text{cuda}, \text{mps}\}$; batch size B; max token length L

Ensure: Each item $r \in \mathcal{J}$ gains: `semantic_similarity_scores`, `semantic_top_score`, `semantic_mean_topk`, `semantic_best_rank`, `semantic_best_chunk_id`

Load tokenizer and model weights from E; move model to d; set model to EVAL mode.
for each JSON file $f \in \mathcal{J}$ **do**
    Load item list $\mathcal{R}$ from f.
    Initialize empty text list $\mathcal{T}$ and deduplication index idx. ▷ single encode pass
    for each item $r \in \mathcal{R}$ that needs update **do**
        $q \leftarrow$ first non-empty of {`explanation_used`, `rationale`, `model_response`, `machine_response`} in r
        Add q to $\mathcal{T}$ if absent; set $idx[q] \leftarrow |\mathcal{T}| - 1$
        for each snippet $h \in r$.`retrieved` **do**
            Add `normalize_ws`(h.`snippet`) to $\mathcal{T}$; record index in idx
        end for
    end for
    for each batch of B texts from $\mathcal{T}$ **do** ▷ no gradient
        Tokenize batch (padding = True, truncation = True, max_length=L)
        Compute $\mathbf{H} \leftarrow$ last hidden state from E
        Mean-pool over token dimension using attention mask: $\mathbf{v} \leftarrow \sum_t \mathbf{H}_t m_t \;/\; \sum_t m_t$
        L2-normalise: $\mathbf{v} \leftarrow \mathbf{v}/\|\mathbf{v}\|_2$
        Store normalised vectors in embedding matrix $\mathbf{Z}$
    end for
    for each item $r \in \mathcal{R}$ that needs update **do**
        $\mathbf{z}_r \leftarrow \mathbf{Z}[idx[q_r]]$ ▷ L2-normalised query embedding
        for each snippet $h_j \in r$.`retrieved` **do**
            $\mathbf{u}_j \leftarrow \mathbf{Z}[idx[h_j.\texttt{snippet}]]$ ▷ L2-normalised snippet embedding
            $s_{rj} \leftarrow \mathbf{z}_r^\top \mathbf{u}_j$ ▷ cosine similarity (vectors are L2-normalised)
        end for
        `semantic_similarity_scores` $\leftarrow [s_{r1}, \ldots, s_{rk}]$
        `semantic_top_score` $\leftarrow \max_j \, s_{rj}$
        `semantic_mean_topk` $\leftarrow \dfrac{1}{k}\sum_{j=1}^{k} s_{rj}$
        `semantic_best_rank` $\leftarrow$ rank of $\arg\max_j s_{rj}$ in r.`retrieved`
        Attach all semantic fields and encoder name to r
    end for
    Write updated $\mathcal{R}$ back to f atomically (temp file, then rename).
end for
▷ **Similarity formula**

$$s_{rj} = \mathbf{z}_r^\top \mathbf{u}_j = \frac{\mathbf{z}_r \cdot \mathbf{u}_j}{\|\mathbf{z}_r\|_2 \, \|\mathbf{u}_j\|_2}, \quad \texttt{semantic\_top\_score} = \max_j \, s_{rj}, \quad \texttt{semantic\_mean\_topk} = \frac{1}{k}\sum_{j=1}^{k} s_{rj}$$

return $\mathcal{J}$ with semantic grounding scores appended to each item
