# Supplementary material for: Retrieve-Then-Verify for Evaluating Evidence Support and Hallucination in Large Language Model–Generated Medical Information: Empirical Study
Source: JMIR AI. 2026 Sep 16;5:e93761. doi: 10.2196/93761 (PMC13628037; doi:10.2196/93761)
Supplement: Supplementary file 6 [file ai_v5i1e93761_app4.pdf]

## Multimedia Appendix 4

### Analysis of semantic similarity by verification verdict and its relationship to retrieval quality

The semantic similarity between LLM rationales and retrieved trial passages was uniformly low, and this raises a key interpretive question. The low similarity may reflect surface-language divergence. In this case the model paraphrases evidence that is in fact present. The low similarity may instead reflect retrieval failure. In this case the retrieved passages do not contain the relevant evidence. The two explanations have different implications. The first explanation concerns how models phrase their reasoning. The second explanation concerns a limitation of the retrieval step. We must distinguish between them to interpret the semantic similarity results in the main text. We therefore analyzed item-level semantic similarity in relation to the verification verdicts and to lexical retrieval strength. If retrieval failure were the main cause, low similarity should concentrate among unsupported verdicts and among items with weak lexical retrieval, and Supported items should show high similarity. Table S1 describes semantic similarity and BM25 retrieval scores by verdict. Table S2 tests whether semantic similarity differs between Supported and non-Supported items. Table S3 quantifies the item-level association between semantic similarity and BM25 retrieval score. Together these analyses test whether low semantic similarity is a marker of retrieval failure or a pervasive feature of model rationales that is largely independent of retrieval quality.

**Table S1. Semantic similarity and BM25 retrieval score by verification verdict**

| Scope / Model | Verdict | n | Max top-k similarity, mean (95% CI) | Mean top-k similarity, mean (95% CI) | BM25 top-1, mean (95% CI) |
|---|---|---|---|---|---|
| All models (pooled) | Supported | 13,264 | 0.49 (0.49–0.49) | 0.42 (0.41–0.42) | 31.46 (31.31–31.62) |
| | Contradicted | 2,078 | 0.47 (0.47–0.48) | 0.41 (0.40–0.41) | 31.56 (31.23–31.89) |
| | Not Found | 5,228 | 0.46 (0.45–0.46) | 0.39 (0.39–0.39) | 30.32 (30.09–30.55) |
| | Out of Scope | 94 | 0.42 (0.40–0.44) | 0.35 (0.34–0.37) | 32.20 (30.18–34.22) |
| GPT-5 | Supported | 4,510 | 0.49 (0.49–0.49) | 0.41 (0.41–0.42) | 31.19 (30.93–31.46) |
| | Contradicted | 614 | 0.47 (0.47–0.48) | 0.41 (0.40–0.41) | 31.49 (30.88–32.11) |
| | Not Found | 1,727 | 0.46 (0.45–0.46) | 0.39 (0.39–0.40) | 30.19 (29.80–30.59) |
| | Out of Scope | 37 | 0.42 (0.39–0.45) | 0.35 (0.33–0.38) | 29.56 (26.86–32.25) |
| GPT-3.5 | Supported | 4,357 | 0.49 (0.49–0.49) | 0.42 (0.41–0.42) | 31.44 (31.17–31.72) |
| | Contradicted | 718 | 0.47 (0.46–0.48) | 0.41 (0.40–0.41) | 31.48 (30.92–32.04) |
| | Not Found | 1,782 | 0.45 (0.45–0.46) | 0.39 (0.39–0.39) | 30.35 (29.96–30.75) |
| | Out of Scope | 31 | 0.42 (0.38–0.45) | 0.36 (0.33–0.39) | 33.13 (29.33–36.94) |
| OpenAI o3-mini | Supported | 4,397 | 0.49 (0.49–0.49) | 0.42 (0.42–0.42) | 31.76 (31.49–32.02) |
| | Contradicted | 746 | 0.48 (0.47–0.48) | 0.41 (0.40–0.42) | 31.70 (31.14–32.25) |
| | Not Found | 1,719 | 0.46 (0.45–0.46) | 0.39 (0.39–0.39) | 30.41 (30.00–30.81) |
| | Out of Scope | 26 | 0.42 (0.39–0.44) | 0.35 (0.33–0.37) | 34.85 (30.80–38.91) |

Table S1 presents the mean maximum top-k cosine similarity and the mean top-k cosine similarity for each verdict. It also reports the mean BM25 top-1 retrieval score for each verdict. The results are shown both pooled across the three models and separately for each model. Semantic similarity declined across verdict categories. The pooled max top-k similarity fell from 0.49 for Supported to 0.47 for Contradicted, 0.46 for Not Found, and 0.42 for Out of Scope. Each model showed the same ordering. The size of this gradient was small and spanned roughly 0.07 across the full range of verdicts. The most important result concerns the Supported verdict. Supported items have confirmed documentary support, yet their absolute similarity was still low (pooled max top-k 0.49; mean top-k 0.42). Retrieval failure would predict high similarity for Supported items. The low similarity for these items therefore indicates that low similarity is a pervasive property of the rationale text rather than a signal of missing evidence. BM25 top-1 scores were comparatively stable across verdicts and ranged from about 30 to 33 across all categories and models. These scores did not decline systematically for unsupported verdicts. Lexical retrieval strength therefore did not differ substantially between supported and unsupported items.

**Table S2. Supported versus non-Supported comparison (Mann-Whitney U test)**

| Scope / Model | Measure | n Supported | n non-Supported | P | Rank-biserial r | Median (Supported) | Median (non-Supported) |
|---|---|---|---|---|---|---|---|
| All models (pooled) | Max top-k similarity | 13,264 | 7,400 | <.001 | 0.17 | 0.49 | 0.46 |
| | Mean top-k similarity | 13,264 | 7,400 | <.001 | 0.14 | 0.42 | 0.40 |
| GPT-5 | Max top-k similarity | 4,510 | 2,378 | <.001 | 0.15 | 0.49 | 0.46 |
| | Mean top-k similarity | 4,510 | 2,378 | <.001 | 0.12 | 0.42 | 0.40 |
| GPT-3.5 | Max top-k similarity | 4,357 | 2,531 | <.001 | 0.18 | 0.49 | 0.46 |
| | Mean top-k similarity | 4,357 | 2,531 | <.001 | 0.14 | 0.42 | 0.40 |
| OpenAI o3-mini | Max top-k similarity | 4,397 | 2,491 | <.001 | 0.17 | 0.49 | 0.47 |
| | Mean top-k similarity | 4,397 | 2,491 | <.001 | 0.14 | 0.42 | 0.40 |

Table S2 tests whether semantic similarity discriminated between Supported items and all non-Supported items. The non-Supported group combined the Contradicted, Not Found, and Out of Scope verdicts. The test was the Mann-Whitney U test, and the rank-biserial correlation served as the effect size. The difference between Supported and non-Supported items was statistically significant in every comparison ($P<.001$). This significance reflects the large item-level sample sizes, which pooled to 13,264 Supported items and 7,400 non-Supported items. The effect size was small throughout. Rank-biserial r ranged from 0.12 to 0.18 across models and metrics, and the pooled values were 0.17 for max top-k and 0.14 for mean top-k. The median similarities also differed only marginally. The pooled max top-k medians were 0.49 for Supported and 0.46 for non-Supported, and the mean top-k medians were 0.42 and 0.40. The results combine statistical significance with a small effect size and near-identical medians. Semantic similarity is therefore weakly associated with verdict, but it does not meaningfully separate supported from unsupported items. This pattern is consistent with surface-language divergence rather than retrieval failure. Missing evidence would produce a substantial separation between supported and unsupported items, but the observed separation was marginal.

**Table S3. Item-level association between semantic similarity and BM25 retrieval score (Spearman rank correlation)**

| Scope / Model | Measure | n | Spearman ρ | P |
|---|---|---|---|---|
| All models (pooled) | Max top-k similarity vs BM25 top-1 | 20,664 | 0.22 | <.001 |
| | Mean top-k similarity vs BM25 top-1 | 20,664 | 0.21 | <.001 |
| GPT-5 | Max top-k similarity vs BM25 top-1 | 6,888 | 0.21 | <.001 |
| | Mean top-k similarity vs BM25 top-1 | 6,888 | 0.20 | <.001 |
| GPT-3.5 | Max top-k similarity vs BM25 top-1 | 6,888 | 0.23 | <.001 |
| | Mean top-k similarity vs BM25 top-1 | 6,888 | 0.22 | <.001 |
| OpenAI o3-mini | Max top-k similarity vs BM25 top-1 | 6,888 | 0.23 | <.001 |
| | Mean top-k similarity vs BM25 top-1 | 6,888 | 0.22 | <.001 |

Table S3 reports the item-level Spearman rank correlation between semantic similarity and BM25 top-1 retrieval score. The results are shown both pooled and per model. The correlations were weak and positive across all configurations. The pooled values were $\rho=0.22$ for max top-k and $\rho=0.21$ for mean top-k, and the per-model values ranged from 0.20 to 0.23. All correlations were statistically significant ($P<.001$) because of the large sample sizes. The weakness of these correlations

indicates that lexical retrieval strength and embedding-based semantic similarity capture largely distinct aspects of evidential grounding. A passage may rank highly under BM25 because it shares terms with the query, yet it may still be phrased differently from the model's rationale. The reverse case can also occur. This independence supports the use of the two metrics as complementary rather than redundant diagnostics. It also argues against the view that low semantic similarity is a simple by-product of weak retrieval, because the two quantities are only loosely coupled at the item level.

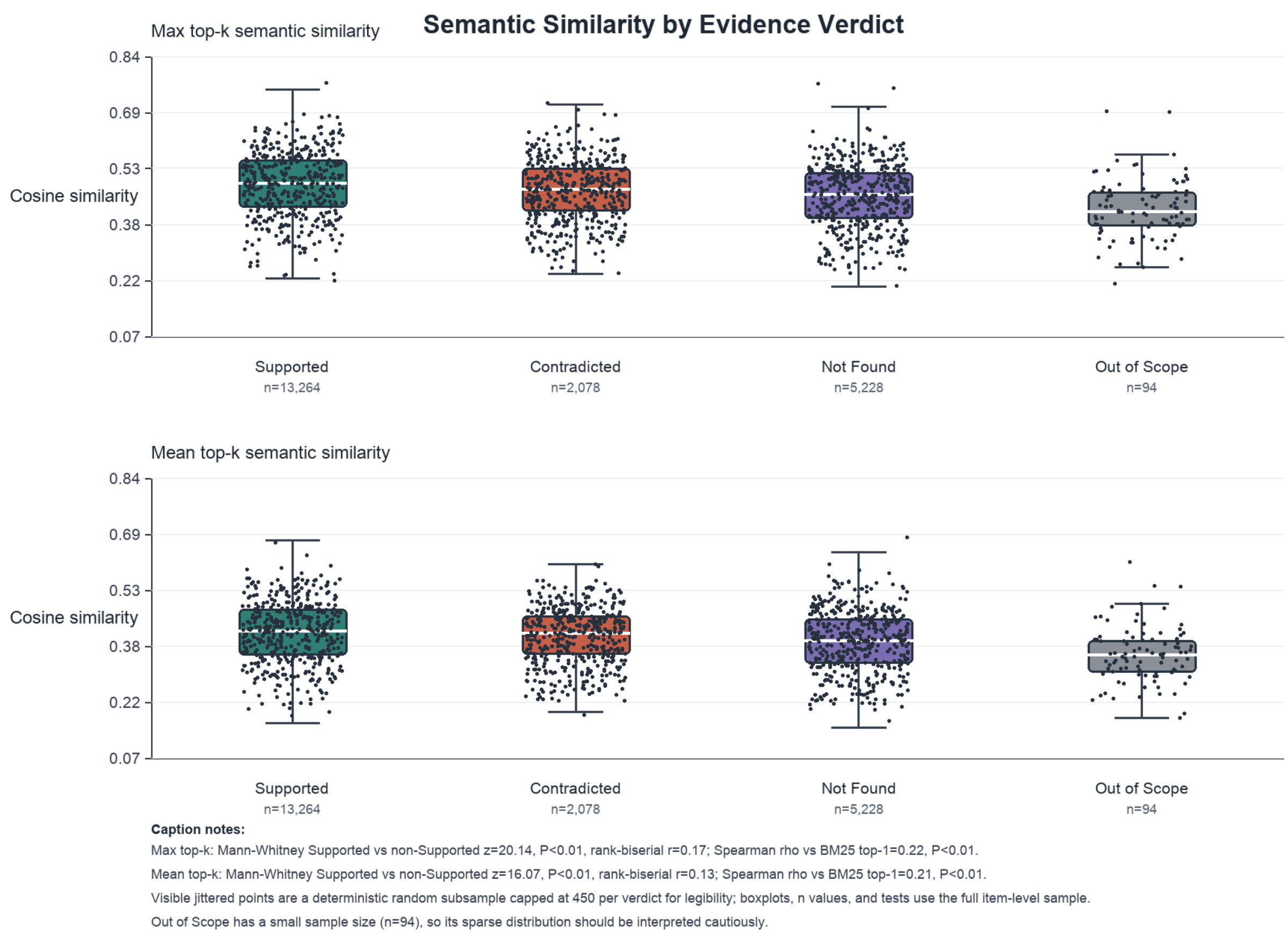


**Figure S1. Distribution of semantic similarity by verification verdict**

Figure S1 displays the distribution of maximum top-k cosine similarity in the upper panel and mean top-k cosine similarity in the lower panel. The distributions are shown for each verification verdict and pooled across the three models. The boxplots

summarize the median and the interquartile range. The overlaid points show a deterministic random subsample of up to 450 items per verdict and improve legibility. The boxplot statistics and all reported tests use the full item-level sample. The figure makes the central finding visually apparent. The four verdict distributions overlap substantially and occupy the same similarity band. The shift from Supported to Out of Scope is only modest. The Supported distribution is centered well below a cosine similarity of 0.5. This confirms that even items with confirmed documentary support have low absolute similarity. The distribution for the Out of Scope category is wide and sparse. This pattern reflects its small sample size (n=94 pooled) and should be interpreted with caution. Figure S1 and Tables S1 to S3 together support one conclusion. Low semantic similarity arises predominantly from systematic differences in surface language between model rationales and source trial text, rather than from failures of the retrieval step. The small verdict gradient indicates that weaker grounding plays only a secondary role. Its effect appears mainly among Not Found and Out of Scope items, where evidence is more often absent or only indirectly related to the claim.
