# Supplementary material for: Retrieve-Then-Verify for Evaluating Evidence Support and Hallucination in Large Language Model–Generated Medical Information: Empirical Study
Source: JMIR AI. 2026 Sep 16;5:e93761. doi: 10.2196/93761 (PMC13628037; doi:10.2196/93761)
Supplement: Supplementary file 7 [file ai_v5i1e93761_app5.pdf]

Algorithm: Quantitatively measure LLM response by BM25 retrieval and LLM Verdicts

Require: Root folders: JSON responses $\mathcal{J}$, PDF root $\mathcal{P}$, output folder $\mathcal{O}$
LLM model (GPT-5) M, OpenAI API key
BM25 params: chunk size L, overlap o, top-k (k=5 by default)
Allowed verdict classes $\mathcal{V} = \{\text{Supported}, \text{Contradicted}, \text{Not Found}, \text{Out of Scope}\}$
Ensure: One verdict JSON per study written to $\mathcal{O}$; global rates:
Support rate $r_{\text{supp}} = \frac{S}{N}$, Conservative hallucination $r_{\text{cons}} = \frac{C+N_f}{N}$, Strict hallucination $r_{\text{strict}} = \frac{C+N_f+O}{N}$
Initialize global counters: $S \leftarrow 0$, $C \leftarrow 0$, $N_f \leftarrow 0$, $O \leftarrow 0$, $N \leftarrow 0$
Set $k \leftarrow 5$ ▷ Default number of retrieved chunks (top-k)
for each study folder $d \in \mathcal{J}$ **do**
  $x_{\text{in}} \leftarrow$ first `.json` file in d; **continue** if none
  $x_{\text{out}} \leftarrow$ path in $\mathcal{O}$ with same basename as x_{in}
  if x_{out} exists and contains a non-empty JSON list **then**
    continue ▷ Skip already-evaluated study
  end if
  $s \leftarrow$ study name (folder basename)
  $p \leftarrow$ first `.pdf` under $\mathcal{P}/s$; **continue** if none
  $\mathcal{R} \leftarrow$ load list of response items from x_{in}
  $T \leftarrow$ `read_pdf`(p)
  $\{t_i\}_{i=1}^{m} \leftarrow$ `chunk`(T, chunk length L, overlap o)
  Build BM25 index B over tokenized chunks $\{t_i\}$
  $\mathcal{E} \leftarrow [\ ]$ ▷ Per-item evaluation records
  for each item $r \in \mathcal{R}$ **do**
    $q \leftarrow$ canonical question text from r.`question_id`
    $y_{\text{model}} \leftarrow r$.`response`; $y_{\text{human}} \leftarrow r$.`human.response`
    $e \leftarrow$ normalize(r.`comment` $\|$ r.`reasoning`)
    ▷ **BM25 query**
    $q^* \leftarrow$ normalize($q \,\|\, y_{\text{model}} \,\|\, e$)
    $\{(t_{i_j}, \sigma_j)\}_{j=1}^{k} \leftarrow \mathsf{B}.\mathtt{topk}(q^*, k)$
    ▷ **LLM verdict**
    Build prompt with (study, question, model/human labels, explanation, retrieved snippets)
    $\hat{v} \leftarrow \mathsf{M}$(prompt) ▷ LLM returns JSON verdict
    $v \leftarrow \mathtt{NormalizeVerdict}(\hat{v}, \mathcal{V})$ ▷ Map to canonical classes
    ▷ **Update tallies**
    $N \leftarrow N + 1$
    if v = Supported **then**
      $S \leftarrow S + 1$
    else if v = Contradicted **then**
      $C \leftarrow C + 1$
    else if v = Not Found **then**
      $N_f \leftarrow N_f + 1$
    else if v = Out of Scope **then**
      $O \leftarrow O + 1$
    end if
    Append to $\mathcal{E}$: { study s, question id, labels, retrieved chunks & scores, verdict v }
  end for
  Write $\mathcal{E}$ as JSON to x_{out}
end for
▷ **Final rates**
$r_{\text{supp}} \leftarrow \dfrac{S}{N}$, $r_{\text{cons}} \leftarrow \dfrac{C + N_f}{N}$, $r_{\text{strict}} \leftarrow \dfrac{C + N_f + O}{N}$
return $r_{\text{supp}}, r_{\text{cons}}, r_{\text{strict}}$ and all per-item outputs
