# Supplementary material for: Retrieve-Then-Verify for Evaluating Evidence Support and Hallucination in Large Language Model–Generated Medical Information: Empirical Study
Source: JMIR AI. 2026 Sep 16;5:e93761. doi: 10.2196/93761 (PMC13628037; doi:10.2196/93761)
Supplement: Supplementary file 8 [file ai_v5i1e93761_app6.pdf]

System prompt: """
You are a reviewer of medical literature. Given a machine response (label), a human response (gold), the exact question asked, and retrieved paper excerpts, decide whether the
machine's claim is Supported, Contradicted, Not Found, or Out of Scope. Return strict JSON only."""

Study: Acarturk 2022
Question ID: 1.2
Question: Was the allocation sequence concealed until participants were enrolled and assigned to interventions?

Machine response (label): Yes
Human (gold) response: Yes

Machine explanation (comment+reasoning):
"Allocation sequence was concealed until enrollment as the research team only accessed the system after participant enrollment. The text indicates that randomization details,
including block sizes, were not available to the recruiters which ensured concealment."

Paper excerpts:
[Chunk 1 | BM25 score=44.67]
"... The audio material imparts key information about stress management and Randomization and masking guides participants ..."
...

Task: Considering the specific question above, judge the machine response relative to the study text.
Answer with this JSON schema only:

```
{
"verdict": "Supported | Contradicted | Not Found | Out of Scope",
"quote": "...",
"rationale": "..."
}
```

Expected LLM response:

```
{
"verdict": "Supported",
"quote": "…Research team members involved in recruitment were able to access the web-based software to randomize each newly enrolled participant, but were not able to access the randomization list, and were not aware of the block size. …",
"rationale": "Centralized, software-based randomization concealed the sequence from recruiters and only allocated after participant information was entered, indicating proper
allocation concealment."
}
```
