# Supplementary material for: Retrieve-Then-Verify for Evaluating Evidence Support and Hallucination in Large Language Model–Generated Medical Information: Empirical Study
Source: JMIR AI. 2026 Sep 16;5:e93761. doi: 10.2196/93761 (PMC13628037; doi:10.2196/93761)
Supplement: Supplementary file 9 [file ai_v5i1e93761_app7.pdf]

Multimedia Appendix 7

This appendix provides a worked example of the Retrieve-Then-Verify (RtV) framework applied to one RCT report (Berger 2018, topic: Anxiety Symptoms 0–1 months). For each RoB 2 signaling question and domain-level conclusion, the entry shows: the AI-generated and human rater responses, the LLM reasoning (explanation_used), BM25 retrieval scores, the top retrieved passages, and the verdict with supporting quote and rationale.

Domain 1 — Bias Arising from the Randomization Process

Question 1.1

Was the allocation sequence random?

AI: **Yes** | Human: **Yes** (match) Verdict: **Supported**

Reasoning: The paper clearly describes using a random procedure (coin toss and dice) to assign participants. Random allocation method was explicitly mentioned, ensuring unbiased allocation.

BM25 top-1: 16.08 | mean top-5: 12.43 | scores: 16.08, 13.94, 11.10, 10.94, 10.08

```
[#1 score=16.08] whether they had experienced a list of physical symptoms in the previous month. Seven somatic symptoms were listed including: stomach, respiratory, headaches, sleeping problems, excessive eating, appetite loss and ‘other problems’. Children answered on a Yes / No categorical scale. This measure was ...
```

```
[#2 score=13.94] l chosen for the study, 183 participated in the study: 95 were randomly assigned to receive the ESPS intervention (the experimental group) and 88 took part in social studies classes (SS control group). Of the 23 students (11.1%) from the six classes whowereexcludedfromthestudy(seeFigure1),9students(...
```

```
[#3 score=11.10] hat students undergoing the ESPS intervention would manifest decreased levels of anxiety, hyperactivity, somatization, and social difficulties and increased prosocial behaviors and functioning at school 826 Transcultural Psychiatry 55(6) compared to students in the active control group. We also pred...
```

Quote: “The principal of this school randomly chose (by tossing a coin) to implement the ESPS (experimental group) in only one of the two classes in each of the 4th–6th grades…”

Rationale: The study explicitly states that allocation to intervention versus control classes was done randomly by coin toss.

Question 1.2

Was the allocation sequence concealed until participants were enrolled and assigned to interventions?

AI: **No information** | Human: **Probably Yes** (differ) Verdict: **Supported**

Reasoning: There is no explicit mention of allocation concealment until participants were enrolled. Details regarding concealment were not provided in the study documentation.

BM25 top-1: 21.18 | mean top-5: 19.11 | scores: 21.18, 19.91, 19.42, 19.19, 15.87

```
[#1 score=21.18] ation of the ESPS intervention There has been growing interest in adapting evidence-based psychological inter- ventions and psychosocial programs originally designed for high and middle- income countries to the needs, priorities and cultural features of communities in LMICs (Barrera & Castro, 2006; ...
```

```
[#2 score=19.91] ervention and were SS control classes and were ESPS intervention and were the SS control classes and not assessed not assessed assessed were assessed Figure 1. Studentflow through therandomization, interventionandassessment protocol. All the parents or guardians whose children were assigned to the E...
```

```
[#3 score=19.42] l chosen for the study, 183 participated in the study: 95 were randomly assigned to receive the ESPS intervention (the experimental group) and 88 took part in social studies classes (SS control group). Of the 23 students (11.1%) from the six classes whowereexcludedfromthestudy(seeFigure1),9students(...
```

Quote: “The principal of this school randomly chose (by tossing a coin) to implement the ESPS (experimental group) in only one of the two classes in each of the 4th–6th grades”

Rationale: The paper describes the randomization method but provides no details on concealing the allocation sequence prior to enrollment or assignment.

Question 1.3

Did baseline differences between intervention groups suggest a problem with the randomization process?

AI: **No** | Human: **No** (match) Verdict: **Not Found**

Reasoning: Baseline differences were checked and no significant differences were found between groups. The groups were comparable at baseline, indicating no issues with randomization.

BM25 top-1: 35.52 | mean top-5: 25.61 | scores: 35.52, 29.85, 23.14, 21.46, 18.10

```
[#1 score=35.52] by the teachers during the academic year for each student.
Adversitywasassessedusinginformationobtainedfromtheschoolregardingthe child’s residence –
child living with family (parent/s or extended family) or child placed in an orphanage,
and the child’s parental loss – parents still alive or either ...
```

```
[#2 score=29.85] ts was found using more objective measures. The ESPS group sig-
nificantlydecreasedthefrequencyofdisciplinarybehaviorsinschoolandimproved
theiracademicperformancecomparedtothecontrolgroup.Moreimportantly,all
thesepositivechangesweresustainedatthe8-monthfollow-up,suggestingthatthe ESPS
intervention w...
```

```
[#3 score=23.14] inaryproblemspriortotheintervention.
Anumberofdifferenceswerealsofoundforgender.Specifically,boysbenefitted to a greater
extent from the intervention in terms of hyperactivity and pro-social behavior than girls.
Again, this finding may be accounted for by the significant gender differences at
baseli...
```

Rationale: The excerpts note that baseline t-tests were performed but do not report the results comparing intervention groups at baseline, so we cannot confirm the absence of problematic baseline differences from the provided text.

Domain 1 Conclusion — Bias Arising from the Randomization Process

Risk-of-bias judgement, based on 1.1–1.3, for bias arising from the randomization process

AI: **Some concerns** | Human: **Some concerns** (match) Verdict: **Supported**

Reasoning: While randomization was conducted and groups were comparable, the lack of reported allocation concealment raises concerns. Random allocation and comparable baselines are strengths, but missing concealment details deem the risk as 'Some concerns'.

BM25 top-1: 26.14 | mean top-5: 23.95 | scores: 26.14, 26.14, 25.04, 21.35, 21.08

```
[#1 score=26.14] ose(bytossingdice)oneschool for the study. The principal of this school
randomly chose (by tossing a coin) to
implementtheESPS(experimentalgroup)inonlyoneofthetwoclassesineachof
the4th-6thgrades,whiletheotherclassesparticipatedinSocialStudies(SS,control
group).Inaddition,theprincipalandteacherspresе...
```

```
[#2 score=26.14] isturbances that threaten system function, viability, or develop- ment’’
(Masten, 2014, p. 6). Nonetheless, there is an ongoing debate as to what constitutes
positive adapta- tionandhowitshouldbedetermined(Bonanno,2012;Masten,2014).Researchers
have suggested that in some contexts, reports of positiv...
```

```
[#3 score=25.04] hat corporal punishment is detrimental to both internalizing and exter-
nalizing behaviors (Hecker, Hermenau, Isele & Elbert, 2014; Feinstein & Berger et al. 839
Mwahombela,2010).Theutilizationofalocalcollectivegriefritualenactedbythe students in one
of the sessions helped the orphans in their class...
```

```
[#4 score=21.35] inaryproblemspriortotheintervention.
Anumberofdifferenceswerealsofoundforgender.Specifically,boysbenefitted to a greater
extent from the intervention in terms of hyperactivity and pro-social behavior than girls.
Again, this finding may be accounted for by the significant gender differences at
baseli...
```

```
[#5 score=21.08] scale derived from the Strengths and Difficulties Questionnaire (SDQ;
Goodman, 1997). This scale is composed of eight items. The children were asked to rate to
what extent each statement described them (e.g. ‘‘I try to be nice to other people. I care
about their feelings’’). All items were ranked on...
```

Quote: “The principal of this school randomly chose (by tossing a coin) to implement the ESPS (experimental group) in only one of the two classes in each of the 4th-6th grades, while the other classes participated in Social Studies (SS, control group).”

Rationale: Randomization occurred but was conducted by the principal with no description of allocation concealment, justifying a 'Some concerns' risk-of-bias judgment for the randomization process.

Domain 2 — Bias Due to Deviations from Intended Interventions

Question 2.1

Were participants aware of their assigned intervention during the trial?

AI: **Yes** | Human: **Probably Yes** (differ) Verdict: **Not Found**

Reasoning: Participants were likely aware of their assigned intervention given the school-based intervention setting. In school settings with distinct intervention activities, participants can easily know their group allocation.

BM25 top-1: 28.95 | mean top-5: 22.17 | scores: 28.95, 23.80, 19.88, 19.27, 18.97

`[#1 score=28.95] hat students undergoing the ESPS intervention would manifest decreased levels of anxiety, hyperactivity, somatization, and social difficulties and increased prosocial behaviors and functioning at school 826 Transcultural Psychiatry 55(6) compared to students in the active control group. We also pred...`

`[#2 score=23.80] ises, (4) eliciting the participa- tion of class members during the session, and (5) students' compliance with homeworkassignments.Mostassessmentswerescoredeither4or5onalldomains and in all classes, and the interrater reliability Kappa value was .76. These assess- ments confirmed high fidelity to th...`

`[#3 score=19.88] ervention and were SS control classes and were ESPS intervention and were the SS control classes and not assessed not assessed assessed were assessed Figure 1. Studentflow through therandomization, interventionandassessment protocol. All the parents or guardians whose children were assigned to the E...`

Rationale: The paper specifies assessor blinding but does not explicitly state whether participants themselves were aware of their assigned intervention, so a definite 'Yes' is not directly supported.

Question 2.2

Were carers and people delivering the interventions aware of participants' assigned intervention during the trial?

AI: **Yes** | Human: **Yes** (match) Verdict: **Supported**

Reasoning: Carers and teachers delivering the intervention were aware of the group assignments. Teachers and involved staff were not blinded in the process; therefore, they knew the interventions administered.

BM25 top-1: 33.99 | mean top-5: 31.65 | scores: 33.99, 32.61, 31.62, 30.62, 29.44

`[#1 score=33.99] l chosen for the study, 183 participated in the study: 95 were randomly assigned to receive the ESPS intervention (the experimental group) and 88 took part in social studies classes (SS control group). Of the 23 students (11.1%) from the six classes whowereexcludedfromthestudy(seeFigure1),9students(...`

`[#2 score=32.61] ises, (4) eliciting the participa- tion of class members during the session, and (5) students' compliance with homeworkassignments.Mostassessmentswerescoredeither4or5onalldomains and in all classes, and the interrater reliability Kappa value was .76. These assess- ments confirmed high fidelity to th...`

`[#3 score=31.62] ose(bytossingdice)oneschool for the study. The principal of this school randomly chose (by tossing a coin) to implementtheESPS(experimentalgroup)inonlyoneofthetwoclassesineachof the4th-6thgrades,whiletheotherclassesparticipatedinSocialStudies(SS,control group).Inaddition,theprincipalandteacherspresese...`

Quote: ""teachers… were trained in delivering the ESPS program to their students" and "the principal and teachers presented the rationale and goals of [the] program to the students' parents or care-takers… and enlisted their support.""

Rationale: Teachers delivering the intervention and parents/caregivers were informed about the program and asked to support homework, indicating they were aware of participants' assigned intervention.

Question 2.3

If 2.1 or 2.2 is Yes/Probably Yes/No information: Were there deviations from the intended intervention that arose because of the trial context?

AI: **No** | Human: **Probably No** (differ) Verdict: **Not Found**

Reasoning: There is no indication that deviations from the intended intervention occurred because of the trial context. Despite lack of blinding, the intervention was implemented with high fidelity and no deviations were reported.

BM25 top-1: 36.61 | mean top-5: 31.75 | scores: 36.61, 35.02, 29.45, 28.86, 28.83

`[#1 score=36.61] ems (i.e. not following the teachers' instructions, disturbing the teachers during class and being disrespectful toward teachers and peers), behavioral problems (i.e. lying, stealing and being verbally and physically aggressive toward peers), somatic com- plaints(i.e.recurrentheadaches,stomachachesw...`

`[#2 score=35.02] whether they had experienced a list of physical symptoms in the previous month. Seven somatic symptoms were listed including: stomach, respiratory, headaches, sleeping problems, excessive eating, appetite loss and 'other problems'. Children answered on a Yes / No categorical scale. This measure was ...`

`[#3 score=29.45] l chosen for the study, 183 participated in the study: 95 were randomly assigned to receive the ESPS intervention (the experimental group) and 88 took part in social studies classes (SS control group). Of the 23 students (11.1%) from the six classes whowereexcludedfromthestudy(seeFigure1),9students(...`

Rationale: The provided excerpts do not report whether any deviations from the intended intervention arose because of the trial context, so the claim of 'No deviations' is not supported by the text.

Question 2.4

If 2.3 is Yes/Probably Yes: Were these deviations likely to have affected the outcome?

AI: **Not Applicable** | Human: **Not Applicable** (match) Verdict: **Not Found**

Reasoning: Since no deviations were detected, the impact on outcomes is not applicable. Without deviations arising from the trial context, assessing their effect is unnecessary.

BM25 top-1: 21.68 | mean top-5: 18.85 | scores: 21.68, 21.45, 18.50, 17.18, 15.47

`[#1 score=21.68] whether they had experienced a list of physical symptoms in the previous month. Seven somatic symptoms were listed including: stomach, respiratory, headaches, sleeping problems, excessive eating, appetite loss and 'other problems'. Children answered on a Yes / No categorical scale. This measure was ...`

`[#2 score=21.45] ts was found using more objective measures. The ESPS group sig- nificantlydecreasedthefrequencyofdisciplinarybehaviorsinschoolandimproved theiracademicperformancecomparedtothecontrolgroup.Moreimportantly,all thesepositivechangesweresustainedatthe8-monthfollow-up,suggestingthatthe ESPS intervention w...`

`[#3 score=18.50] me concepts that were particularly applicable to the Tanzanian culture such as respecting the elderly (shikamoo) and providing mutual support (ujamaais). Additionally, local popular folktalesandtraditionalfablessuchasthe''theValueofaPerson'';''TheCalabash Kids'' and ''The Tortoise and the Hare'' (An...`

Rationale: The excerpts do not report whether deviations from intended interventions occurred or whether they were likely to affect outcomes, so the applicability of this question cannot be determined from the text.

Question 2.5

If 2.4 is Yes/Probably Yes/No information: Were these deviations from intended intervention balanced between groups?

AI: **Not Applicable** | Human: **Not Applicable** (match) Verdict: **Not Found**

Reasoning: There were no deviations to be balanced between groups. No deviations occurred, so balance between groups is not a concern.

BM25 top-1: 22.14 | mean top-5: 19.69 | scores: 22.14, 20.76, 20.56, 19.53, 15.48

`[#1 score=22.14] whether they had experienced a list of physical symptoms in the previous month. Seven somatic symptoms were listed including: stomach, respiratory, headaches, sleeping problems, excessive eating, appetite loss and 'other problems'. Children answered on a Yes / No categorical scale. This measure was ...`

`[#2 score=20.76] ts was found using more objective measures. The ESPS group sig- nificantlydecreasedthefrequencyofdisciplinarybehaviorsinschoolandimproved`

theiracademicperformancecomparedtothecontrolgroup.Moreimportantly,all thesepositivechangesweresustainedatthe8-monthfollow-up,suggestingthatthe ESPS intervention w...

[#3 score=20.56] ervention and were SS control classes and were ESPS intervention and were the SS control classes and not assessed not assessed assessed were assessed Figure 1. Studentflow through therandomization, interventionandassessment protocol. All the parents or guardians whose children were assigned to the E...

Rationale: The provided excerpts do not report on any deviations from the intended intervention or whether such deviations were balanced between groups, so the 'Not Applicable' claim cannot be verified.

Question 2.6

Was an appropriate analysis used to estimate the effect of assignment to intervention?

AI: **Yes** | Human: **No information** (differ) Verdict: **Not Found**

Reasoning: An appropriate analysis (repeated measures MANOVA and ANOVAs) was used to estimate the effect of assignment. The study used standard statistical methods that are suitable for a randomized controlled trial design.

BM25 top-1: 48.12 | mean top-5: 34.10 | scores: 48.12, 38.21, 28.80, 28.28, 27.08

[#1 score=48.12] by the teachers during the academic year for each student. Adversitywasassessedusinginformationobtainedfromtheschoolregardingthe child’s residence – child living with family (parent/s or extended family) or child placed in an orphanage, and the child’s parental loss – parents still alive or either ...

[#2 score=38.21] ts was found using more objective measures. The ESPS group sig- nificantlydecreasedthefrequencyofdisciplinarybehaviorsinschoolandimproved theiracademicperformancecomparedtothecontrolgroup.Moreimportantly,all thesepositivechangesweresustainedatthe8-monthfollow-up,suggestingthatthe ESPS intervention w...

[#3 score=28.80] s X group; Wilk’s Lambda¼.32; F¼11.58; df¼27, 178; p<.001). Thereafter, repeated measures ANOVAs were performed for each independent measure. The ESPS accounted for significant changes on all dependent measures immediately after the interven- tion. These effects were sustained 8 months later (see Ta...

Rationale: While the paper reports using repeated-measures MANOVA/ANOVAs, it does not state whether analyses were conducted by assignment (e.g., intention-to-treat) or otherwise appropriate for estimating the effect of assignment, so adequacy cannot be determined from the excerpts.

Question 2.7

If 2.6 is No/Probably No/No information: Was there potential for a substantial impact of failure to analyse participants in their randomized groups?

AI: **Not Applicable** | Human: **Probably No** (differ) Verdict: **Not Found**

Reasoning: Since an appropriate analysis was used, there is no potential impact from analysis failure. No issues with analysis mean that concerns about analytic impact are not applicable.

BM25 top-1: 40.87 | mean top-5: 28.59 | scores: 40.87, 26.51, 26.47, 25.28, 23.83

[#1 score=40.87] ts was found using more objective measures. The ESPS group sig- nificantlydecreasedthefrequencyofdisciplinarybehaviorsinschoolandimproved theiracademicperformancecomparedtothecontrolgroup.Moreimportantly,all thesepositivechangesweresustainedatthe8-monthfollow-up,suggestingthatthe ESPS intervention w...

[#2 score=26.51] by the teachers during the academic year for each student. Adversitywasassessedusinginformationobtainedfromtheschoolregardingthe child’s residence – child living with family (parent/s or extended family) or child placed in an orphanage, and the child’s parental loss – parents still alive or either ...

[#3 score=26.47] ems (i.e. not following the teachers’ instructions, disturbing the teachers during class and being disrespectful toward teachers and peers), behavioral problems (i.e. lying, stealing and being verbally and physically aggressive toward peers), somatic com- plaints(i.e.recurrentheadaches,stomachachesw...

Rationale: The excerpts describe statistical methods but do not mention randomization or analysis by randomized groups, so there is no textual basis to deem the concern not applicable.

Domain 2 Conclusion — Bias Due to Deviations from Intended Interventions

Risk-of-bias judgement, based on 2.1–2.7, for deviations from intended interventions (effect of assignment)

AI: **Low risk** | Human: **Low risk** (match) Verdict: **Supported**

Reasoning: Although participants and providers were aware of the assignments, there were no deviations and the analysis was appropriate. The non-blinding did not lead to bias via deviations, which supports a low risk judgment for this domain.

BM25 top-1: 31.97 | mean top-5: 28.96 | scores: 31.97, 28.81, 28.59, 27.92, 27.51

```
[#1 score=31.97] ts was found using more objective measures. The ESPS group sig-
nificantlydecreasedthefrequencyofdisciplinarybehaviorsinschoolandimproved
theiracademicperformancecomparedtothecontrolgroup.Moreimportantly,all
thesepositivechangesweresustainedatthe8-monthfollow-up,suggestingthatthe ESPS
intervention w...
```

```
[#2 score=28.81] -Gunn, J. (Eds.). (1997). Consequences of growing up poor. New York,
NY:Russell SageFoundation.
Durlak,J.A.,Weissberg,R.P.,Dymnicki,A.B.,Taylor,R.D.,&Schellinger,K.B.(2011). The impact
of enhancing students’ social and emotional learning: A meta-analysis of school-based
universal interventions. Chil...
```

```
[#3 score=28.59] ose(bytossingdice)oneschool for the study. The principal of this school
randomly chose (by tossing a coin) to
implementtheESPS(experimentalgroup)inonlyoneofthetwoclassesineachof
the4th-6thgrades,whiletheotherclassesparticipatedinSocialStudies(SS,control
group).Inaddition,theprincipalandteacherspresе...
```

```
[#4 score=27.92] heselfandothersandtoencouragethemtoactpro-sociallyintheschoolandthe
community. The stress-reduction topics covered include understanding the nature
ofstressreactions,strengtheningnaturalinnerresources,calmingthebody,coping with fear,
anger, loss, and grief, building social support and developing an ...
```

```
[#5 score=27.51] hat corporal punishment is detrimental to both internalizing and exter-
nalizing behaviors (Hecker, Hermenau, Isele & Elbert, 2014; Feinstein & Berger et al. 839
Mwahombela,2010).Theutilizationofalocalcollectivegriefritualenactedbythe students in one
of the sessions helped the orphans in their class...
```

Quote: “Although this study involved randomized classes, an active control group, a manualized protocol... and a fidelity assessment”

Rationale: Delivery followed a manual with fidelity monitoring and no reported deviations due to awareness of assignment, supporting a low risk for deviations from intended interventions (effect of assignment).

Domain 3 — Bias Due to Missing Outcome Data

Question 3.1

Were data for this outcome available for all, or nearly all, participants randomized?

AI: **Probably Yes** | Human: **Probably Yes** (match) Verdict: **Not Found**

Reasoning: Data were available for a high proportion (approximately 89%) of participants. Despite some exclusions, the retention rate is high enough to consider data availability nearly complete.

BM25 top-1: 25.35 | mean top-5: 21.62 | scores: 25.35, 24.45, 21.03, 20.08, 17.21

```
[#1 score=25.35] tional, and social development, and for this reason is an ideal context
to address their socioemotional needs (Fazel, Hoagood, Stephan & Ford, 2014).
Implementing universal interventions within the school system helps make these services
more available, feasible and affordable to a relatively large ...
```

```
[#2 score=24.45] ), suspension for several days, and sometimes referring them to local
healers or giving them local 830 Transcultural Psychiatry 55(6) herbal medicine. However,
they also indicated that most of these measures where either partially effective or not
effective at all. Based on this information, the ori...
```

[#3 score=21.03] ervention and were SS control classes and were ESPS intervention and were the SS control classes and not assessed not assessed assessed were assessed Figure 1. Studentflow through therandomization, interventionandassessment protocol. All the parents or guardians whose children were assigned to the E...

Rationale: The provided excerpts do not report the proportion of randomized participants with outcome data or attrition details, so data completeness cannot be determined.

Question 3.2

If 3.1 is No/Probably No/No information: Is there evidence that the result was not biased by missing outcome data?

AI: **Not Applicable** | Human: **Not Applicable** (match) Verdict: **Not Found**

Reasoning: Since nearly all data were available, there is no further concern about bias due to missing data. High retention minimizes the risk of bias from missing outcome data, making further analysis unnecessary.

BM25 top-1: 30.64 | mean top-5: 26.20 | scores: 30.64, 29.72, 25.46, 23.82, 21.37

[#1 score=30.64]), suspension for several days, and sometimes referring them to local healers or giving them local 830 Transcultural Psychiatry 55(6) herbal medicine. However, they also indicated that most of these measures where either partially effective or not effective at all. Based on this information, the ori...

[#2 score=29.72] ts was found using more objective measures. The ESPS group sig- nificantlydecreasedthefrequencyofdisciplinarybehaviorsinschoolandimproved theiracademicperformancecomparedtothecontrolgroup.Moreimportantly,all thesepositivechangesweresustainedatthe8-monthfollow-up,suggestingthatthe ESPS intervention w...

[#3 score=25.46] whether they had experienced a list of physical symptoms in the previous month. Seven somatic symptoms were listed including: stomach, respiratory, headaches, sleeping problems, excessive eating, appetite loss and ‘other problems’. Children answered on a Yes / No categorical scale. This measure was ...

Rationale: The provided excerpts do not report retention, attrition, or handling of missing outcome data to justify deeming the question Not Applicable.

Question 3.3

If 3.2 is No/Probably No: Could missingness in the outcome depend on its true value?

AI: **Not Applicable** | Human: **Not Applicable** (match) Verdict: **Not Found**

Reasoning: Missing data are minimal, so the dependency of missingness on true values is not a concern. With the nearly complete dataset, potential dependency of missingness on outcome values is not relevant.

BM25 top-1: 28.58 | mean top-5: 26.17 | scores: 28.58, 27.03, 27.01, 25.98, 22.26

[#1 score=28.58] whether they had experienced a list of physical symptoms in the previous month. Seven somatic symptoms were listed including: stomach, respiratory, headaches, sleeping problems, excessive eating, appetite loss and ‘other problems’. Children answered on a Yes / No categorical scale. This measure was ...

[#2 score=27.03] ts was found using more objective measures. The ESPS group sig- nificantlydecreasedthefrequencyofdisciplinarybehaviorsinschoolandimproved theiracademicperformancecomparedtothecontrolgroup.Moreimportantly,all thesepositivechangesweresustainedatthe8-monthfollow-up,suggestingthatthe ESPS intervention w...

[#3 score=27.01] land, which matched the amount of time spent on the ESPS intervention. These lessons were also provided by the homeroom teachers and were delivered in the control classes at the same time as the ESPS intervention. The contents included topics such as the history of the country, political issues, civ...

Rationale: The excerpts provide no information on the extent or pattern of missing outcome data, so we cannot assess applicability or whether missingness could depend on the true outcome.

Question 3.4

If 3.3 is Yes/Probably Yes/No information: Is it likely that missingness in the outcome depended on its true value?

AI: **Not Applicable** | Human: **Not Applicable** (match) Verdict: **Not Found**

Reasoning: Not applicable due to the minimal amount of missing data. The low rate of missing data negates the need to consider its dependency on the true values.

BM25 top-1: 41.20 | mean top-5: 34.84 | scores: 41.20, 34.64, 34.00, 33.63, 30.75

```
[#1 score=41.20] whether they had experienced a list of physical symptoms in the previous month. Seven somatic symptoms were listed including: stomach, respiratory, headaches, sleeping problems, excessive eating, appetite loss and ‘other problems’. Children answered on a Yes / No categorical scale. This measure was ...
```

```
[#2 score=34.64] scale derived from the Strengths and Difficulties Questionnaire (SDQ; Goodman, 1997). This scale is composed of eight items. The children were asked to rate to what extent each statement described them (e.g. ‘‘I try to be nice to other people. I care about their feelings’’). All items were ranked on...
```

```
[#3 score=34.00] me concepts that were particularly applicable to the Tanzanian culture such as respecting the elderly (shikamoo) and providing mutual support (ujamaais). Additionally, local popular folktalesandtraditionalfablessuchasthe‘‘theValueofaPerson’’;‘‘TheCalabash Kids’’ and ‘‘The Tortoise and the Hare’’ (An...
```

Rationale: The provided excerpts do not mention missing outcome data or its dependence on true values, so there is no textual basis to judge the applicability.

Domain 3 Conclusion — Bias Due to Missing Outcome Data

Risk-of-bias judgement, based on 3.1–3.4, for missing outcome data

AI: **Low risk** | Human: **Low risk** (match) Verdict: **Not Found**

Reasoning: Overall, missing outcome data are minimal and unlikely to bias the study results. High data availability and minimal exclusions result in a low risk of bias from missing outcome data.

BM25 top-1: 28.66 | mean top-5: 26.53 | scores: 28.66, 28.26, 25.95, 25.22, 24.57

```
[#1 score=28.66] ), suspension for several days, and sometimes referring them to local healers or giving them local 830 Transcultural Psychiatry 55(6) herbal medicine. However, they also indicated that most of these measures where either partially effective or not effective at all. Based on this information, the ori...
```

```
[#2 score=28.26] hat corporal punishment is detrimental to both internalizing and exter- nalizing behaviors (Hecker, Hermenau, Isele & Elbert, 2014; Feinstein & Berger et al. 839 Mwahombela,2010).Theutilizationofalocalcollectivegriefritualenactedbythe students in one of the sessions helped the orphans in their class...
```

```
[#3 score=25.95] e randomly assigned to either the 16-session ‘‘ERSAE-Stress-Prosocial (ESPS)’’ structured intervention or to a Social Study curriculum (SS) active control group. The original ESPS program was adapted by Tanzanians mental health professionals who modified the program based on local idioms of distress...
```

```
[#4 score=25.22] equally effective at different adversity levels as well as gender cate- gories, descriptive statistics at Time 1 and group comparisons were calculated. Thereafter,repeatedmeasuresANOVAswithonlytheparticipantsintheinterven- tion group were computed for all outcome measures. All analyses were conducte...
```

```
[#5 score=24.57] -Gunn, J. (Eds.). (1997). Consequences of growing up poor. New York, NY:Russell SageFoundation. Durlak,J.A.,Weissberg,R.P.,Dymnicki,A.B.,Taylor,R.D.,&Schellinger,K.B.(2011). The impact of enhancing students’ social and emotional learning: A meta-analysis of school-based universal interventions. Chil...
```

Rationale: The provided excerpts do not report attrition, completeness of follow-up, or handling of missing data, so a low risk-of-bias judgment for missing outcome data cannot be verified.

Domain 4 — Bias in Measurement of the Outcome

Question 4.1

Was the method of measuring the outcome inappropriate?

AI: **No** | Human: **No** (match) Verdict: **Supported**

Reasoning: The measurement methods, including validated questionnaires and objective records, are appropriate. Standard and reliable instruments were used, indicating no inappropriateness in measurement.

BM25 top-1: 24.54 | mean top-5: 20.85 | scores: 24.54, 22.84, 20.90, 18.15, 17.80

[#1 score=24.54] ons. In particular, the sample was recruited from one primary school; thus, our results may not be generalizable to other settings. Second, since the students in the experimental and control groups attended the same school, some contamination effects cannot be ruled out. Although the current study e...

[#2 score=22.84] ts was found using more objective measures. The ESPS group sig-nificantlydecreasedthefrequencyofdisciplinarybehaviorsinschoolandimproved theiracademicperformancecomparedtothecontrolgroup.Moreimportantly,all thesepositivechangesweresustainedatthe8-monthfollow-up,suggestingthatthe ESPS intervention w...

[#3 score=20.90] whether they had experienced a list of physical symptoms in the previous month. Seven somatic symptoms were listed including: stomach, respiratory, headaches, sleeping problems, excessive eating, appetite loss and ‘other problems’. Children answered on a Yes / No categorical scale. This measure was ...

Quote: “This measure was derived from the Diagnostic Predictive Scales (DISC/DPS; Lucas et al, 2001), which has been shown to be valid and reliable.”

Rationale: The study used validated, reliable instruments and standard objective records, indicating appropriate outcome measurement despite acknowledged potential bias from unblinded teachers.

Question 4.2

Could measurement or ascertainment of the outcome have differed between intervention groups?

AI: **Probably Yes** | Human: **Probably No** (differ) Verdict: **Supported**

Reasoning: Measurement of certain outcomes (e.g., disciplinary records) could differ between groups due to non-blinded assessors. Teacher-reported outcomes may be influenced by their awareness of group assignments, leading to potential differential measurement.

BM25 top-1: 27.10 | mean top-5: 23.23 | scores: 27.10, 25.21, 21.46, 21.38, 21.02

[#1 score=27.10] ESPS intervention appears to have been equally effective for students who experienced different levels of adversities. However, theinterventionwasfoundtobemoreeffectiveinimprovingacademicachievement amongchildrenlivinginanorphanagethanchildrenwhowerelivingathome.This finding may be attributed to th...

[#2 score=25.21] ons. In particular, the sample was recruited from one primary school; thus, our results may not be generalizable to other settings. Second, since the students in the experimental and control groups attended the same school, some contamination effects cannot be ruled out. Although the current study e...

[#3 score=21.46] e randomly assigned to either the 16-session ‘‘ERSAE-Stress-Prosocial (ESPS)’’ structured intervention or to a Social Study curriculum (SS) active control group. The original ESPS program was adapted by Tanzanians mental health professionals who modified the program based on local idioms of distress...

Quote: “the objective measures may have been biased since they were completed by the teachers who were not blind to the intervention.”

Rationale: Non-blinded teachers collected some outcomes, creating potential for differential measurement between intervention groups.

Question 4.3

If 4.1 and 4.2 are No/Probably No/No information: Were outcome assessors aware of the intervention received?

AI: **Yes** | Human: **Probably Yes** (differ) Verdict: **Supported**

Reasoning: Outcome assessors, particularly teachers reporting disciplinary measures, were aware of the intervention received. The involvement of non-blinded teachers in data collection on some outcomes qualifies as assessors being aware of the interventions.

BM25 top-1: 33.45 | mean top-5: 30.87 | scores: 33.45, 32.54, 29.88, 29.29, 29.18

[#1 score=33.45] ts was found using more objective measures. The ESPS group sig-nificantlydecreasedthefrequencyofdisciplinarybehaviorsinschoolandimproved

theiracademicperformancecomparedtothecontrolgroup.Moreimportantly,all thesepositivechangesweresustainedatthe8-monthfollow-up,suggestingthatthe ESPS intervention w...

[#2 score=32.54] ons. In particular, the sample was recruited from one primary school; thus, our results may not be generalizable to other settings. Second, since the students in the experimental and control groups attended the same school, some contamination effects cannot be ruled out. Although the current study e...

[#3 score=29.88] ation of the ESPS intervention There has been growing interest in adapting evidence-based psychological inter- ventions and psychosocial programs originally designed for high and middle- income countries to the needs, priorities and cultural features of communities in LMICs (Barrera & Castro, 2006; ...

Quote: "the objective measures may have been biased since they were completed by the teachers who were not blind to the intervention."

Rationale: The paper explicitly states teachers completing objective outcomes were not blinded, indicating assessors were aware of the intervention.

Question 4.4

If 4.3 is Yes/Probably Yes/No information: Could assessment of the outcome have been influenced by knowledge of intervention received?

AI: **Probably Yes** | Human: **Probably Yes** (match) Verdict: **Supported**

Reasoning: It is likely that knowledge of the intervention could have influenced the assessment of outcomes measured by the teachers. The non-blinding of certain assessors introduces the possibility of bias in outcome assessment.

BM25 top-1: 37.13 | mean top-5: 34.58 | scores: 37.13, 35.33, 34.21, 33.14, 33.10

[#1 score=37.13] whether they had experienced a list of physical symptoms in the previous month. Seven somatic symptoms were listed including: stomach, respiratory, headaches, sleeping problems, excessive eating, appetite loss and 'other problems'. Children answered on a Yes / No categorical scale. This measure was ...

[#2 score=35.33] hat corporal punishment is detrimental to both internalizing and exter- nalizing behaviors (Hecker, Hermenau, Isele & Elbert, 2014; Feinstein & Berger et al. 839 Mwahombela,2010).Theutilizationofalocalcollectivegriefritualenactedbythe students in one of the sessions helped the orphans in their class...

[#3 score=34.21] ESPS intervention appears to have been equally effective for students who experienced different levels of adversities. However, theinterventionwasfoundtobemoreeffectiveinimprovingacademicachievement amongchildrenlivinginanorphanagethanchildrenwhowerelivingathome.This finding may be attributed to th...

Quote: ""These lessons were also provided by the homeroom teachers…" and "…the ESPS group significantly decreased the frequency of disciplinary behaviors in school and improved their academic performance compared to the control group.""

Rationale: Because homeroom teachers delivered the interventions and outcomes included teacher-influenced measures (disciplinary behavior and academic performance), knowledge of allocation could plausibly influence outcome assessment.

Question 4.5

If 4.4 is Yes/Probably Yes/No information: Is it likely that assessment of the outcome was influenced by knowledge of intervention received?

AI: **Probably Yes** | Human: **Probably No** (differ) Verdict: **Supported**

Reasoning: It is likely that teacher assessments, being aware of group allocation, may have been influenced by this knowledge. Given that teachers knew which students received the intervention, their evaluations could be positively or negatively biased.

BM25 top-1: 32.28 | mean top-5: 28.45 | scores: 32.28, 28.42, 27.81, 27.26, 26.47

[#1 score=32.28] tial exercises were changed. For instance, in session 5A (see Box 1), instead of employing the Western cognitive-behavioral framework for dealing with ''automatic negative thoughts'' that was part of the original ESPS manual (Berger et al., 2015), the teachers took a local indigenous perspective tha...

[#2 score=28.42] nfluenceacademicachievementas well as social interactions (Fredrickson, 2001; Nelson, 2009). Future research is needed to explore the mechanisms underlying these interventions as well as their relative contribution. Altogether, given the cumulative

risk factors that Tanzanian and other LMIC children...

[#3 score=27.81] ESPS intervention appears to have been equally effective for students who experienced different levels of adversities. However, theinterventionwasfoundtobemoreeffectiveinimprovingacademicachievement amongchildrenlivinginanorphanagethanchildrenwhowerelivingathome.This finding may be attributed to th...

Quote: “the objective measures may have been biased since they were completed by the teachers who were not blind to the intervention.”

Rationale: Teachers were unblinded and completed some outcome measures, making influence of knowledge of intervention on outcome assessment likely.

Domain 4 Conclusion — Bias in Measurement of the Outcome

Risk-of-bias judgement, based on 4.1–4.5, for measurement of the outcome

AI: **Some concerns** | Human: **Some concerns** (match) Verdict: **Supported**

Reasoning: While measurement methods were mostly appropriate, non-blinded teacher assessments raise some concerns. The mixed use of blinded self-reports and non-blinded teacher reports leads to some risk of bias in outcome measurement.

BM25 top-1: 33.11 | mean top-5: 28.19 | scores: 33.11, 32.42, 26.15, 25.19, 24.09

[#1 score=33.11] isturbances that threaten system function, viability, or develop- ment’’ (Masten, 2014, p. 6). Nonetheless, there is an ongoing debate as to what constitutes positive adapta- tionandhowitshouldbedetermined(Bonanno,2012;Masten,2014).Researchers have suggested that in some contexts, reports of positiv...

[#2 score=32.42] ons. In particular, the sample was recruited from one primary school; thus, our results may not be generalizable to other settings. Second, since the students in the experimental and control groups attended the same school, some contamination effects cannot be ruled out. Although the current study e...

[#3 score=26.15] hat students undergoing the ESPS intervention would manifest decreased levels of anxiety, hyperactivity, somatization, and social difficulties and increased prosocial behaviors and functioning at school 826 Transcultural Psychiatry 55(6) compared to students in the active control group. We also pred...

[#4 score=25.19] parental losses, physical and sexual abuse and armed conflicts, which put them at high risk of developing a wide range of emotional and behavioral problems, it is imperative to help them develop resiliency skills. Therefore, the goal of this study was to facilitate Tanzanian students’ resiliency thr...

[#5 score=24.09] 388–398. Fazel,M.,Reed,R.V.,Panter-Brick,C.,&Stein,A.(2012).Mentalhealthofdisplacedand refugee children resettled in high-income countries: risk and protective factors. The Lancet,379(9812), 266–282. Feinstein, S., & Mwahombela, L. (2010). Corporal punishment in Tanzania’s schools. InternationalRevi...

Quote: “the objective measures may have been biased since they were completed by the teachers who were not blind to the intervention.”

Rationale: Non-blinded teacher assessments risk biased outcome measurement, consistent with a 'Some concerns' judgment.

Domain 5 — Bias in Selection of the Reported Result

Question 5.1

Were the data that produced this result analysed in accordance with a pre-specified analysis plan finalized before unblinded outcome data were available?

AI: **No information** | Human: **Probably Yes** (differ) Verdict: **Supported**

Reasoning: There is no mention of a pre-specified analysis plan or pre-registration. The study did not provide details indicating that the analysis followed a pre-specified protocol.

BM25 top-1: 28.76 | mean top-5: 26.87 | scores: 28.76, 28.68, 28.51, 24.27, 24.13

[#1 score=28.76] by the teachers during the academic year for each student. Adversitywasassessedusinginformationobtainedfromtheschoolregardingthe child’s residence – child living with family (parent/s or extended family) or child placed in an orphanage, and the child’s parental loss – parents still alive or either ...

[#2 score=28.68] ts was found using more objective measures. The ESPS group sig-
nificantlydecreasedthefrequencyofdisciplinarybehaviorsinschoolandimproved
theiracademicperformancecomparedtothecontrolgroup.Moreimportantly,all
thesepositivechangesweresustainedatthe8-monthfollow-up,suggestingthatthe ESPS
intervention w...

[#3 score=28.51] l chosen for the study, 183 participated in the study: 95 were randomly assigned to receive the ESPS intervention (the experimental group) and 88 took part in social studies classes (SS control group). Of the 23 students (11.1%) from the six classes whowereexcludedfromthestudy(seeFigure1),9students(...

Quote: “Statistical analyses Descriptive statistics for all outcome measures at baseline were calculated... To assess the effect of the intervention over time, a repeated measure MANOVA...”

Rationale: The paper describes the analyses performed but gives no indication of a pre-specified analysis plan or registration finalized before unblinded outcome data were available.

Question 5.2

Is the numerical result likely to have been selected from multiple eligible outcome measurements (scales/definitions/time points) within the domain?

AI: **Probably No** | Human: **Probably No** (match) Verdict: **Supported**

Reasoning: The outcomes were consistently reported without signs of selective outcome measurement. Although multiple outcomes were measured, the reporting appears comprehensive without selective emphasis.

BM25 top-1: 29.15 | mean top-5: 21.69 | scores: 29.15, 21.00, 19.99, 19.62, 18.68

[#1 score=29.15] ts was found using more objective measures. The ESPS group sig-
nificantlydecreasedthefrequencyofdisciplinarybehaviorsinschoolandimproved
theiracademicperformancecomparedtothecontrolgroup.Moreimportantly,all
thesepositivechangesweresustainedatthe8-monthfollow-up,suggestingthatthe ESPS
intervention w...

[#2 score=21.00] whether they had experienced a list of physical symptoms in the previous month. Seven somatic symptoms were listed including: stomach, respiratory, headaches, sleeping problems, excessive eating, appetite loss and ‘other problems’. Children answered on a Yes / No categorical scale. This measure was ...

[#3 score=19.99] ons. In particular, the sample was recruited from one primary school; thus, our results may not be generalizable to other settings. Second, since the students in the experimental and control groups attended the same school, some contamination effects cannot be ruled out. Although the current study e...

Quote: “Figure 2. Prosocial behavior before, after and 8 months after the intervention.”

Rationale: The study used specific standard scales for each outcome and reported results across all time points, indicating no selective choice among multiple measurements within a domain.

Question 5.3

Is the numerical result likely to have been selected from multiple eligible analyses of the data?

AI: **Probably No** | Human: **Probably No** (match) Verdict: **Supported**

Reasoning: The analyses were clearly described and there is no clear evidence of selective analytical practices. The reporting of results suggests a straightforward analysis without data dredging.

BM25 top-1: 26.04 | mean top-5: 25.18 | scores: 26.04, 25.73, 25.04, 24.76, 24.35

[#1 score=26.04] ts was found using more objective measures. The ESPS group sig-
nificantlydecreasedthefrequencyofdisciplinarybehaviorsinschoolandimproved
theiracademicperformancecomparedtothecontrolgroup.Moreimportantly,all
thesepositivechangesweresustainedatthe8-monthfollow-up,suggestingthatthe ESPS
intervention w...

[#2 score=25.73] whether they had experienced a list of physical symptoms in the previous month. Seven somatic symptoms were listed including: stomach, respiratory, headaches, sleeping problems, excessive eating, appetite loss and ‘other problems’. Children answered on a Yes / No categorical scale. This measure was ...

[#3 score=25.04]), suspension for several days, and sometimes referring them to local healers or giving them local 830 Transcultural Psychiatry 55(6) herbal medicine. However, they also indicated that most of these measures where either partially effective or not effective at all. Based on this information, the ori...

Quote: “To assess the effect of the intervention over time, a repeated measure MANOVA including all outcome measures was conducted... The MANOVA analysis was significant and thus was followed by separate repeated measure ANOVAs for each outcome measure.”

Rationale: The study describes a clear, specified analysis plan with standard follow-ups, reducing the likelihood that results were selected from multiple eligible analyses.

Domain 5 Conclusion — Bias in Selection of the Reported Result

Risk-of-bias judgement, based on 5.1–5.3, for selection of the reported result

AI: **Low risk** | Human: **Low risk** (match) Verdict: **Supported**

Reasoning: The risk of bias in selection of the reported result is low as the reporting appears complete. There is no strong evidence of selective reporting based on the comprehensive presentation of results.

BM25 top-1: 30.33 | mean top-5: 29.64 | scores: 30.33, 29.80, 29.72, 29.71, 28.63

[#1 score=30.33] e randomly assigned to either the 16-session ‘‘ERSAE-Stress-Prosocial (ESPS)’’ structured intervention or to a Social Study curriculum (SS) active control group. The original ESPS program was adapted by Tanzanians mental health professionals who modified the program based on local idioms of distress...

[#2 score=29.80] ts was found using more objective measures. The ESPS group sig- nificantlydecreasedthefrequencyofdisciplinarybehaviorsinschoolandimproved theiracademicperformancecomparedtothecontrolgroup.Moreimportantly,all thesepositivechangesweresustainedatthe8-monthfollow-up,suggestingthatthe ESPS intervention w...

[#3 score=29.72] inaryproblemspriortotheintervention. Anumberofdifferenceswerealsofoundforgender.Specifically,boysbenefitted to a greater extent from the intervention in terms of hyperactivity and pro-social behavior than girls. Again, this finding may be accounted for by the significant gender differences at baseli...

[#4 score=29.71] olence, and almost three-quarters report experiencing physical violence in childhood (UNICEF, 2011). A large body of research has shown that exposure to cumulative adversity in childhood dramatically heightens the risk of developing long-term mental, emotional and behavioral problems in highly devel...

[#5 score=28.63] tional, and social development, and for this reason is an ideal context to address their socioemotional needs (Fazel, Hoagood, Stephan & Ford, 2014). Implementing universal interventions within the school system helps make these services more available, feasible and affordable to a relatively large ...

Quote: “Students’ resilience was evaluated before, after and 8 months following the intervention by assessing social difficulties, hyperactivity, somatization, level of anxiety, prosocial behaviors and school functioning as well as academic achievements and disciplinary problems. There was significant improvement on all outcome measures for the ESPS group compared to the control group post-intervention and at the 8-month follow up.”

Rationale: The study reports multiple predefined outcomes across all time points with comprehensive results, consistent with a low risk of selective reporting.

Overall Risk-of-Bias Judgment

Based on all domains, assess the overall risk of bias

AI: **Some concerns** | Human: **Some concerns** (match) Verdict: **Supported**

Reasoning: Overall, while many domains show low risk, concerns in allocation concealment and outcome measurement lead to an overall judgment of some concerns. The combination of solid randomization and analysis with gaps in concealment and some measurement bias results in an overall 'Some concerns' rating.

BM25 top-1: 39.25 | mean top-5: 32.79 | scores: 39.25, 32.77, 31.94, 30.53, 29.46

[#1 score=39.25] ises, (4) eliciting the participa- tion of class members during the session, and (5) students’ compliance with homeworkassignments.Mostassessmentswerescoredeither4or5onalldomains and in all classes,

and the interrater reliability Kappa value was .76. These assess- ments confirmed high fidelity to th...

[#2 score=32.77] hat corporal punishment is detrimental to both internalizing and exter- nalizing behaviors (Hecker, Hermenau, Isele & Elbert, 2014; Feinstein & Berger et al. 839 Mwahombela,2010).Theutilizationofalocalcollectivegriefritualenactedbythe students in one of the sessions helped the orphans in their class...

[#3 score=31.94] isturbances that threaten system function, viability, or develop- ment’’ (Masten, 2014, p. 6). Nonetheless, there is an ongoing debate as to what constitutes positive adapta- tionandhowitshouldbedetermined(Bonanno,2012;Masten,2014).Researchers have suggested that in some contexts, reports of positiv...

[#4 score=30.53] ons. In particular, the sample was recruited from one primary school; thus, our results may not be generalizable to other settings. Second, since the students in the experimental and control groups attended the same school, some contamination effects cannot be ruled out. Although the current study e...

[#5 score=29.46] inaryproblemspriortotheintervention. Anumberofdifferenceswerealsofoundforgender.Specifically,boysbenefitted to a greater extent from the intervention in terms of hyperactivity and pro-social behavior than girls. Again, this finding may be accounted for by the significant gender differences at baseli...

Quote: ““some contamination effects cannot be ruled out… the objective measures may have been biased since they were completed by the teachers who were not blind to the intervention.””

Rationale: The study explicitly notes potential contamination and unblinded outcome assessment, indicating risk of bias consistent with an overall judgment of some concerns.

BM25 retrieval: chunk_size=1,400 chars, overlap=200 chars, top-k=5. Judge model: GPT-5. Study: Berger 2018, topic: Anxiety Symptoms (0–1 months).
