# Supplementary material for: Retrieve-Then-Verify for Evaluating Evidence Support and Hallucination in Large Language Model–Generated Medical Information: Empirical Study
Source: JMIR AI. 2026 Sep 16;5:e93761. doi: 10.2196/93761 (PMC13628037; doi:10.2196/93761)
Supplement: Supplementary file 10 [file ai_v5i1e93761_app8.pdf]

Multimedia Appendix 8

Effect of generator-model reasoning depth on evidence support and hallucination (GPT-5 minimal vs medium reasoning)

The judge-model comparison examines how the reasoning capacity of the verifier affects verdict assignment. However, it does not address a separate question: how the reasoning depth of the generator model affects its own tendency to produce unsupported claims. This appendix reports a supplementary analysis of this question.

We re-generated all RoB assessments with GPT-5 at minimal reasoning and evaluated them with the same RtV pipeline used for the default medium-reasoning condition. Only the reasoning level of the generator model was changed. The 97 studies, the document chunking parameters, the BM25 retrieval settings, the prompt templates, and the GPT-5 medium-reasoning judge were all held constant across the two conditions, and both conditions used the same model snapshot. This design isolates the reasoning depth of the generator model as the single varied factor.

Table S4. Evidence support and hallucination by generator-model reasoning condition

Topic	Supp % (Medium)	Supp % (Minimal)	Δ Supp	Cons hall % (Medium)	Cons hall % (Minimal)	Δ Cons	Strict hall % (Medium)	Strict hall % (Minimal)	Δ Strict
Pooled	65.48	55.92	−9.55	33.99	42.07	+8.09	34.52	44.08	+9.55
Adverse events	50.00	64.29	+14.29	41.07	33.93	−7.14	50.00	35.71	−14.29
Anxiety symptoms	62.80	51.64	−11.16	36.76	47.32	+10.57	37.20	48.36	+11.16
Depressive symptoms	63.38	56.69	−6.69	36.07	41.20	+5.13	36.62	43.31	+6.69
Diagnosis of mental disorders	67.19	60.49	−6.70	31.92	37.05	+5.13	32.81	39.51	+6.70
Distress/PTSD symptoms	68.04	57.02	−11.02	31.66	41.40	+9.75	31.96	42.98	+11.02

Topic	Supp % (Medium)	Supp % (Minimal)	Δ Supp	Cons hall % (Medium)	Cons hall % (Minimal)	Δ Cons	Strict hall % (Medium)	Strict hall % (Minimal)	Δ Strict
Psychological functioning and impairment	63.10	56.35	−6.75	36.31	40.87	+4.56	36.91	43.65	+6.75
Quality of life	70.16	54.26	−15.90	29.26	43.09	+13.83	29.84	45.74	+15.90
Social outcomes	64.00	53.43	−10.57	35.86	44.57	+8.71	36.00	46.57	+10.57

Note: Δ denotes minimal minus medium. Supp %, Cons hall %, and Strict hall % are the support rate, conservative hallucination rate, and strict hallucination rate. Paired comparisons across the eight topics (Wilcoxon signed-rank test) gave P=.15 for support, P=.05 for conservative hallucination, and P=.15 for strict hallucination; the mean paired topic differences were −6.81, +6.32, and +6.81, respectively. The Adverse events topic is based on a single study (n=56) and should be interpreted with caution.

Table S5. Task accuracy by generator-model reasoning condition

Topic	Exact acc % (Medium)	Exact acc % (Minimal)	Δ Exact	Binary acc % (Medium)	Binary acc % (Minimal)	Δ Binary	Binary Acc–Support Gap (Medium)	Binary Acc–Support Gap (Minimal)
Pooled	55.87	44.60	−11.27	89.49	77.65	−11.84	24.01	21.73
Adverse events	60.71	51.79	−8.93	94.74	87.88	−6.86	44.74	23.59
Anxiety symptoms	56.12	44.18	−11.94	90.08	75.00	−15.08	27.28	23.36
Depressive symptoms	53.05	45.69	−7.36	88.60	78.42	−10.18	25.22	21.73
Diagnosis of mental disorders	51.23	42.28	−8.95	91.89	79.08	−12.81	24.70	18.59
Distress/PTSD symptoms	59.06	46.52	−12.54	89.23	77.82	−11.41	21.19	20.79
Psychological functioning and impairment	59.96	45.22	−14.74	90.03	78.02	−12.01	26.94	21.67
Quality of life	56.93	45.96	−10.97	89.92	79.41	−10.52	19.76	25.14
Social outcomes	54.43	36.14	−18.29	89.23	73.19	−16.04	25.23	19.76

Note: Δ denotes minimal minus medium. Exact acc %, Binary acc % are the exact accuracy and binary accuracy. Exact accuracy is the proportion of outputs that exactly matched the human label; binary accuracy is computed after collapsing ordinal categories to decision-level correctness. The Binary Accuracy–Support Gap is binary accuracy minus the support rate

Table S6. Retrieval and semantic grounding diagnostics by generator-model reasoning condition

Topic	BM25 (Medium)	BM25 (Minimal)	Δ BM25	Semantic max (Medium)	Semantic max (Minimal)	Δ max	Semantic mean (Medium)	Semantic mean (Minimal)	Δ mean
Pooled	30.96	28.49	−2.48	0.35	0.37	+0.02	0.26	0.27	+0.01
Adverse events	23.60	29.98	+6.38	0.40	0.37	−0.03	0.26	0.28	+0.01
Anxiety symptoms	30.43	28.16	−2.28	0.34	0.37	+0.04	0.24	0.27	+0.03
Depressive symptoms	30.41	28.40	−2.01	0.35	0.37	+0.03	0.26	0.28	+0.02
Diagnosis of mental disorders	30.91	28.36	−2.56	0.33	0.35	+0.02	0.24	0.26	+0.02
Distress/PTSD symptoms	29.77	27.65	−2.12	0.35	0.36	+0.01	0.26	0.27	+0.01
Psychological functioning and impairment	30.64	28.61	−2.03	0.36	0.37	+0.01	0.26	0.28	+0.01
Quality of life	33.75	28.63	−5.12	0.36	0.36	0.00	0.27	0.27	0.00
Social outcomes	33.26	30.72	−2.54	0.36	0.37	+0.01	0.26	0.27	+0.01

Note: Δ denotes minimal minus medium. BM25 is the retrieval score of the highest-ranked passage. Semantic max and mean are the maximum and mean cosine similarity between the LLM explanation and the retrieved passages, computed with the sentence-transformers/all-MiniLM-L6-v2 encoder using L2-normalized embeddings.

Data Interpretation

Reducing the reasoning depth of the generator model lowered evidence support and raised hallucination. Pooled evidence support fell from 65.48% to 55.92%, a decrease of 9.55 percentage-point. Strict hallucination rose from 34.52% to 44.08% by the same amount. The direction was consistent across seven of the eight topics. The only exception was the topic of adverse events, which is based on a single study (n=56) and is the topic for which we advise caution throughout. The paired topic-level tests did not reach significance for support (P=.15), which reflects the small number of topics and the single reversal rather than an absence of effect.

Reducing reasoning depth also lowered task accuracy. Pooled binary accuracy fell from 89.49% to 77.65%, a decrease of 11.84 percentage points, and exact accuracy fell from 55.87% to 44.60%. Binary accuracy decreased in all eight topics. This is the most consistent signal in the analysis. The decrease in binary accuracy was similar in size to the decrease in evidence support. As a result, the binary Accuracy–Support Gap remained close to its medium-reasoning value at the pooled level (24.01 to 21.73). This pattern differs from the main finding of the study. In the main analysis, high decision accuracy coexisted with weaker evidence grounding. Here, lower reasoning depth reduced decision accuracy and evidence grounding together. The dissociation between accuracy and grounding therefore appears to depend on the model's reasoning capacity, and it narrows when that capacity is removed.

The degradation was not an artifact of retrieval. Mean BM25 top-1 scores were only slightly lower under minimal reasoning (30.96 to 28.49, a decrease of 2.48). This small change is unlikely to account for a support drop of about 10 percentage points. The minor BM25 decrease is most likely due to shorter explanation text under minimal reasoning, which made the retrieval query less rich. Semantic similarity did not decrease under minimal reasoning. The maximum top-k similarity rose slightly from 0.35 to 0.37, and the mean top-k similarity rose from 0.26 to 0.27. Evidence support fell sharply while semantic similarity barely moved. This result is consistent with the finding in Multimedia Appendix 10 that semantic similarity is a weak grounding signal and does not track documentary support.

The shift in verdicts shows how grounding degraded. Of the items that left the Supported category under minimal reasoning, the pooled counts moved into Contradicted (614 to 914), Not Found (1,727 to 1,984), and Out of Scope (37 to 138). The Out of Scope count nearly quadrupled. Minimal reasoning therefore produced both more contradicted claims and more claims that could not be grounded in the retrieved text.

These results indicate that the reasoning depth of the generator model is an important determinant of both its task accuracy and its evidence grounding. The analysis used a single alternative reasoning level on one systematic review. A full characterization across multiple reasoning levels, models, and clinical domains remains a direction for future work.
