# Supplementary material for: Retrieve-Then-Verify for Evaluating Evidence Support and Hallucination in Large Language Model–Generated Medical Information: Empirical Study
Source: JMIR AI. 2026 Sep 16;5:e93761. doi: 10.2196/93761 (PMC13628037; doi:10.2196/93761)
Supplement: Supplementary file 11 [file ai_v5i1e93761_app9.pdf]

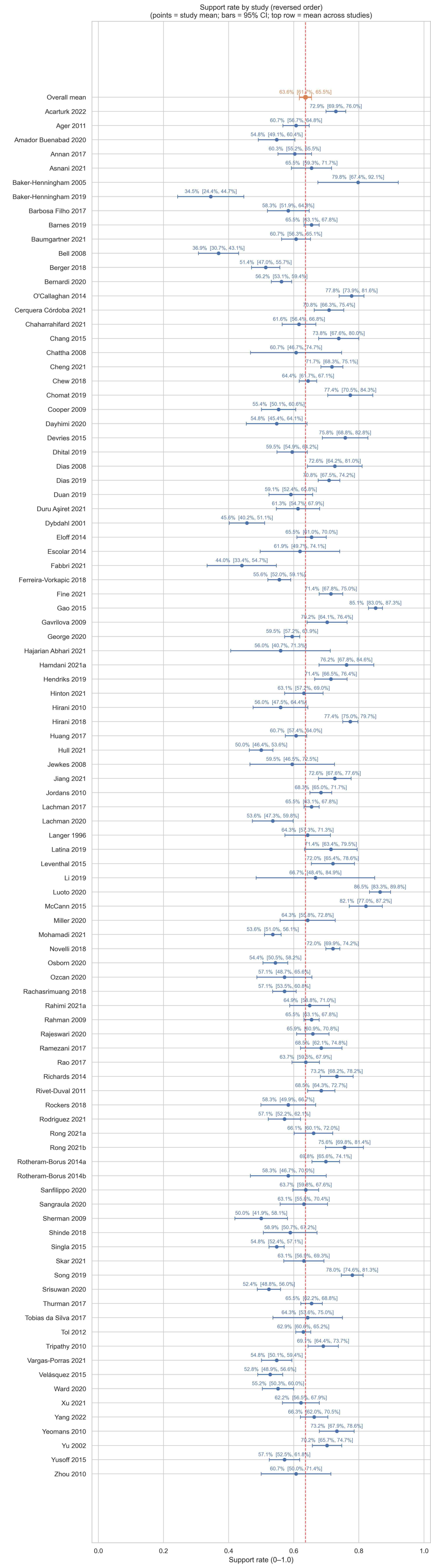
Support rate by study (reversed order)
(points = study mean; bars = 95% CI; top row = mean across studies)
Overall mean
63.6% [61.7%, 65.5%]
Acarturk 2022
72.9% [69.9%, 76.0%]
Ager 2011
60.7% [56.7%, 64.8%]
Amador Buenabad 2020
54.8% [49.1%, 60.4%]
Annan 2017
60.3% [55.2%, 65.5%]
Asnani 2021
65.5% [59.3%, 71.7%]
Baker-Henningham 2005
79.8% [67.4%, 92.1%]
Baker-Henningham 2019
34.5% [24.4%, 44.7%]
Barbosa Filho 2017
58.3% [51.9%, 64.8%]
Barnes 2019
65.5% [63.1%, 67.8%]
Baumgartner 2021
60.7% [56.3%, 65.1%]
Bell 2008
36.9% [30.7%, 43.1%]
Berger 2018
51.4% [47.0%, 55.7%]
Bernardi 2020
56.2% [53.1%, 59.4%]
O'Callaghan 2014
77.8% [73.9%, 81.6%]
Cerquera Córdoba 2021
70.8% [66.3%, 75.4%]
Chaharrahifard 2021
61.6% [56.4%, 66.8%]
Chang 2015
73.8% [67.6%, 80.0%]
Chattha 2008
60.7% [46.7%, 74.7%]
Cheng 2021
71.7% [68.3%, 75.1%]
Chew 2018
64.4% [61.7%, 67.1%]
Chomat 2019
77.4% [70.5%, 84.3%]
Cooper 2009
55.4% [50.1%, 60.6%]
Dayhimi 2020
54.8% [45.4%, 64.1%]
Devries 2015
75.8% [68.8%, 82.8%]
Dhital 2019
59.5% [54.9%, 64.2%]
Dias 2008
72.6% [64.2%, 81.0%]
Dias 2019
70.8% [67.5%, 74.2%]
Duan 2019
59.1% [52.4%, 65.8%]
Duru Aşiret 2021
61.3% [54.7%, 67.9%]
Dybdahl 2001
45.6% [40.2%, 51.1%]
Eloff 2014
65.5% [61.0%, 70.0%]
Escolar 2014
61.9% [49.7%, 74.1%]
Fabbri 2021
44.0% [33.4%, 54.7%]
Ferreira-Vorkapic 2018
55.6% [52.0%, 59.1%]
Fine 2021
71.4% [67.8%, 75.0%]
Gao 2015
85.1% [83.0%, 87.3%]
Gavrilova 2009
70.2% [64.1%, 76.4%]
George 2020
59.5% [57.2%, 61.9%]
Hajarian Abhari 2021
56.0% [40.7%, 71.3%]
Hamdani 2021a
76.2% [67.8%, 84.6%]
Hendriks 2019
71.4% [66.5%, 76.4%]
Hinton 2021
63.1% [57.2%, 69.0%]
Hirani 2010
56.0% [47.5%, 64.4%]
Hirani 2018
77.4% [75.0%, 79.7%]
Huang 2017
60.7% [57.4%, 64.0%]
Hull 2021
50.0% [46.4%, 53.6%]
Jewkes 2008
59.5% [46.5%, 72.5%]
Jiang 2021
72.6% [67.6%, 77.6%]
Jordans 2010
68.3% [65.0%, 71.7%]
Lachman 2017
65.5% [63.1%, 67.8%]
Lachman 2020
53.6% [47.3%, 59.8%]
Langer 1996
64.3% [57.3%, 71.3%]
Latina 2019
71.4% [63.4%, 79.5%]
Leventhal 2015
72.0% [65.4%, 78.6%]
Li 2019
66.7% [48.4%, 84.9%]
Luoto 2020
86.5% [83.3%, 89.8%]
McCann 2015
82.1% [77.0%, 87.2%]
Miller 2020
64.3% [55.8%, 72.8%]
Mohamadi 2021
53.6% [51.0%, 56.1%]
Novelli 2018
72.0% [69.9%, 74.2%]
Osborn 2020
54.4% [50.5%, 58.2%]
Ozcan 2020
57.1% [48.7%, 65.6%]
Rachasrimuang 2018
57.1% [53.5%, 60.8%]
Rahimi 2021a
64.9% [58.8%, 71.0%]
Rahman 2009
65.5% [63.1%, 67.8%]
Rajeswari 2020
65.9% [60.9%, 70.8%]
Ramezani 2017
68.5% [62.1%, 74.8%]
Rao 2017
63.7% [59.5%, 67.9%]
Richards 2014
73.2% [68.2%, 78.2%]
Rivet-Duval 2011
68.5% [64.3%, 72.7%]
Rockers 2018
58.3% [49.9%, 66.7%]
Rodriguez 2021
57.1% [52.2%, 62.1%]
Rong 2021a
66.1% [60.1%, 72.0%]
Rong 2021b
75.6% [69.8%, 81.4%]
Rotheram-Borus 2014a
69.8% [65.6%, 74.1%]
Rotheram-Borus 2014b
58.3% [46.7%, 70.0%]
Sanfilippo 2020
63.7% [59.8%, 67.6%]
Sangraula 2020
63.1% [55.8%, 70.4%]
Sherman 2009
50.0% [41.9%, 58.1%]
Shinde 2018
58.9% [50.7%, 67.2%]
Singla 2015
54.8% [52.4%, 57.1%]
Skar 2021
63.1% [56.9%, 69.3%]
Song 2019
78.0% [74.6%, 81.3%]
Srisuwan 2020
52.4% [48.8%, 56.0%]
Thurman 2017
65.5% [62.2%, 68.8%]
Tobias da Silva 2017
64.3% [53.6%, 75.0%]
Tol 2012
62.9% [60.6%, 65.2%]
Tripathy 2010
69.1% [64.4%, 73.7%]
Vargas-Porras 2021
54.8% [50.1%, 59.4%]
Velásquez 2015
52.8% [48.9%, 56.6%]
Ward 2020
55.2% [50.3%, 60.0%]
Xu 2021
62.2% [56.5%, 67.9%]
Yang 2022
66.3% [62.0%, 70.5%]
Yeomans 2010
73.2% [67.9%, 78.6%]
Yu 2002
70.2% [65.7%, 74.7%]
Yusoff 2015
57.1% [52.5%, 61.8%]
Zhou 2010
60.7% [50.0%, 71.4%]
0.0
0.2
0.4
0.6
0.8
1.0
Support rate (0–1.0)
